# A generalizable covalent car-t platform for solid tumors enabled by oncolytic adenovirus-delivered artificial antigens

**DOI:** 10.3389/fimmu.2026.1919208

**Published:** 2026-08-26

**Authors:** Linpei Guo, Hui Xie, Di Yin, Guidong Zhu, Peichao Zhai, GuoJing Pei, Wen Zhang, Fang Xiao, Jiaju Lyu, Dongqi Tang

**Affiliations:** 1 Center for Gene and Immunotherapy, The Second Qilu Hospital of Shandong University, Jinan, China; 2 Department of Urology, The First Affiliated Hospital of USTC, Division of Life Sciences and Medicine, University of Science and Technology of China, Hefei, China; 3 Department of Radiology, The First Affiliated Hospital of Shandong First Medical University& Shandong Provincial Qianfoshan Hospital, Jinan, China; 4 Department of Neurosurgery, The Second Qilu Hospital of Shandong University, Jinan, China; 5 Department of Urology, The Second Qilu Hospital of Shandong University, Jinan, China; 6 Institute of Medical Sciences, The Second Qilu Hospital of Shandong University, Jinan, China; 7 Multidisciplinary Innovation Center for Nephrology of the Second Qilu Hospital of Shandong University, Jinan, China; 8 Department of Gerontology, The Second Qilu Hospital of Shandong University, Jinan, China; 9 Department of Urology, Shandong Provincial Hospital Affiliated to Shandong First Medical University, Jinan, China

**Keywords:** artificial antigens, chimeric antigen receptor T cell (CAR T cell), covalent CAR-T, oncolytic adenoviruses (OAds), SpyCatcher/SpyTag

## Abstract

**Background:**

Chimeric antigen receptor (CAR) T-cell therapy has shown limited efficacy against solid tumors due to antigen heterogeneity and scarcity of tumor-specific targets.

**Methods:**

To address those challenges, we report a covalent CAR-T strategy that achieves programmable tumor recognition via oncolytic adenovirus-mediated (OAD) delivery of artificial antigens. An engineered OAD was designed to induce tumor-selective expression of a membrane-anchored SpyTag-containing artificial antigen on infected tumor cells. In parallel, we generated SpyCatcher CAR-T cells by replacing the conventional single-chain variable fragment (scFv) with SpyCatcher, which forms a spontaneous covalent bond with SpyTag and redirects CAR-T-cell activity toward virus-labeled tumor cells.

**Results:**

*In vitro*, optimized SpyCatcher CAR-T cells mediated selective cytotoxicity against SpyTag-positive tumor cells, achieving >85% specific lysis at an effector-to-target ratio of 1:1, while sparing antigen-negative cells. The OAD efficiently induced tumor-selective expression of membrane-anchored SpyTag-fused antigens across multiple cell lines. Combined treatment with OAD and SpyCatcher CAR-T cells resulted in substantially greater antitumor activity than either monotherapy alone. *In vivo*, the combinatorial strategy significantly inhibited tumor growth and increased intratumor CD3^+^, CD8^+^ T-cell infiltration in both immunodeficient and immunocompetent mouse models. Importantly, patient-derived prostate cancer organoids were effectively transduced by OAD and supported robust SpyCatcher CAR-T cell infiltration and cytotoxicity, demonstrating the translational potential of this approach.

**Conclusions:**

This study establishes a modular platform for solid tumor immunotherapy therapy by integrating covalent SpyCatcher CAR-T cells with SpyTag-delivering OAD. By decoupling tumor recognition from endogenous antigen expression, this approach provides a generalizable strategy to overcome antigen heterogeneity and scarcity of tumor-specific targets in solid tumors.

## Introduction

Chimeric antigen receptor T (CAR-T) cell therapy has marked the advent of targeted cellular drugs. In hematological malignancies, CAR-T therapy targeting CD19 has achieved remarkable success, significantly improving complete remission rates and long-term survival (1–3). However, the application of CAR-T cells in solid tumors remains challenging owing to the lack of tumor-specific antigens (TSAs) in most tumor types (4–7). The inability of immune cells to efficiently recognize tumor cells hinders the identification of ideal CAR T targets. Furthermore, the high heterogeneity of tumor cells and immune evasion mechanisms contribute to the gradual reduction or even loss of tumor immunogenicity during tumor progression, preventing the immune system from effectively recognizing tumor cells (8–13). Consequently, immunotherapy and CAR-T therapy for solid tumors are limited by several challenges, including antigen heterogeneity, immunosuppressive tumor microenvironment, and severe off-target effects (14–18). To overcome these limitations, the development of a strategy to deliver highly specific “non-self” markers into tumors would greatly expand the potential of targeted therapy and CAR-T applications in solid tumors (19–21).

Oncolytic viruses (OVs) are genetically engineered viruses designed to selectively infect and lyse tumor cells. Several OVs, such as T-VEC, G47δ, and Adstiladrin, have been approved for the treatment of melanoma, glioblastoma, and bladder cancer, respectively, demonstrating established safety profiles (22–24). OVs are excellent vehicles for tumor-targeted delivery, eliciting immune responses within tumors (25, 26). By carrying therapeutic genes, OVs can infect and lyse tumor cells, releasing tumor antigens and therapeutic proteins to trigger antitumor immunity (27, 28). Additionally, OVs engineered to express cytokines and immune checkpoint inhibitors can further modulate the tumor microenvironment and enhance antitumor immunity (29, 30).

The SpyCatcher/SpyTag system is a versatile and robust protein ligation technology derived from *Streptococcus pyogenes* fibronectin-binding protein (31–33). It consists of the SpyCatcher protein, which contains a catalytic domain, and the SpyTag peptide, which has a short 13-amino-acid sequence. Upon contact, SpyTag and SpyCatcher spontaneously form isopeptide bonds between specific lysine and aspartate residues, resulting in rapid and irreversible covalent conjugation. This system has been widely adopted in biotechnology for protein engineering, vaccine development, molecular assembly, and more recently, in the design of targeted therapeutic agents, owing to its high efficiency, specificity, and modular applicability (34–37).

In this study, we propose a tumor cell labeling strategy to introduce a highly specific “non-self” marker onto tumor cells. Our “label-and-kill” strategy comprises (1) labeling tumor cells using a modified oncolytic adenovirus (SpyTag OAD) and (2) recognizing and eliminating labeled tumor cells using CAR-T cells engineered with SpyCatcher (SpyCatcher CAR-T). This approach leverages the SpyCatcher/SpyTag system, in which the SpyCatcher protein covalently binds with high specificity to the SpyTag peptide. First, we used tumor-targeting OAD to express SpyTag fusion proteins on the surfaces of tumor cells. Subsequently, SpyCatcher CAR-T cells specifically recognize the labeled tumor cells via stable covalent interactions between SpyCatcher and SpyTag, leading to targeted tumor cell elimination. This study aimed to establish a universal strategy for treating solid tumors by integrating SpyTag OAD with SpyCatcher CAR-T cells.

## Materials and methods

### Patient samples and tissue collection

Prostate cancer tissues were obtained from patients undergoing radical prostatectomy at the Second Qilu Hospital of Shandong University. The specimens used in this study were residual clinical samples collected after routine pathological diagnosis, and no additional tissue was obtained solely for research purposes. Written informed consent was obtained from all patients for the use of their residual surgical specimens for research purposes and publication. The study was approved by the Institutional Review Board of the Second Qilu Hospital of Shandong University (approval no. KYLL202602076) and conducted in accordance with the Declaration of Helsinki. None of the patients had received prior hormonal therapy, chemotherapy, or radiotherapy before surgery. Fresh tissue was collected from pathologist-confirmed tumor-rich regions identified by hematoxylin and eosin (H&E) staining. Specimens were immediately placed in ice-cold Advanced DMEM/F12 (Gibco, 12634010) supplemented with 1× penicillin-streptomycin and transported to the laboratory within 2 hours for processing.

### DNA constructs

Relevant DNA sequences were synthesized by Genewiz and subsequently inserted into the expression vector via In-Fusion cloning. All the constructed plasmids were verified by Sanger sequencing.

### Western blotting

Total cellular proteins were extracted using radio-immunoprecipitation assay lysis buffer, and the supernatant was collected after centrifugation at 13,000 ×*g* for 30 min. The proteins were heated at 95 °C for 5 min in loading buffer, and 30 μg of protein was loaded per well. After separation using sodium dodecyl sulfate-polyacrylamide gel electrophoresis (SDS-PAGE), the proteins were transferred onto a polyvinylidene fluoride membranes. The membrane was blocked with 5% skim milk and then incubated with primary antibodies overnight at 4 °C on a shaker. After incubation with secondary antibodies at room temperature for 1 h, the bands were visualized using enhanced chemiluminescence (ECL) detection reagent.

### Prokaryotic expression of recombinant protein

The target gene was cloned into the pET-28b (+) vector and sequenced by Sanger sequencing. The plasmid was transformed into chemically competent cells. A single colony was selected and inoculated into lysogeny broth (LB) medium for overnight culture. The overnight culture was diluted 1:100 into fresh LB medium, and when the OD_600_ reached 0.6, protein expression was induced by adding 0.5 mM isopropyl β-d-1-thiogalactopyranoside. The culture was incubated at 20 °C with shaking at 200 rpm for 24 h. The bacterial cells were harvested by centrifugation and lysed using a high-pressure homogenizer. The lysate was centrifuged to collect the supernatant, which was subjected to Ni-affinity chromatography to purify the target protein. The buffer was then replaced with phosphate-buffered saline (PBS) via ultrafiltration. Protein purity was assessed using SDS-PAGE.

### SpyCatcher/SpyTag binding assay in cells

Plasmids encoding SpyCatcher or SpyTag were transfected into target cells using Lipofectamine 3000. The cells were harvested after 48 h of incubation. Approximately 1 × 10^6^ transfected cells were resuspended in 100 μL fluorescent activated cell sorting (FACS) buffer and incubated on ice for 30 min with varying concentrations of SpyTag-sfGFP (superfolder green fluorescent protein) or SpyCatcher-sfGFP protein, along with anti-Myc-APC (allophycocyanin) antibody. Binding was analyzed using flow cytometry.

For imaging-based detection, the transfected target cells were seeded onto glass coverslips. After adherence, the cells were incubated with 5 μM SpyTag-sfGFP or SpyCatcher-sfGFP and anti-Myc-APC antibodies at room temperature for 15 min. Nuclei were stained with Hoechst 33342 for 10 min and observed under a fluorescence microscope.

### Lentiviral packaging, purification, and titer determination

Lentiviral-expressing plasmids were co-transfected with the packaging plasmid psPAX2 and envelope plasmid pMD2.G into 293T cells using polyethylenimine (PEI) reagent. After 8 h, the medium was replaced with a fresh culture medium. Supernatants were collected 24, 48, and 72 h post-transfection. The collected supernatants were centrifuged and filtered through a 0.45-μm membrane to remove cell debris. Lentiviral particles were precipitated overnight using PEG8000 and pelleted by centrifugation. The pellet was resuspended in PBS and stored at −80 °C.

For titration, Jurkat cells were seeded in 96-well plates at 1 × 10^4^ cells per well in 100 μL of culture medium. The lentivirus was then serially diluted 10-fold. After 48 h of incubation, GFP-positive cells were quantified using flow cytometry to determine the viral titer for subsequent T cell transduction.

### Human T cell isolation, lentiviral transduction, and ex vivo expansion

Leukapheresis was obtained from consenting research participants (healthy donors) under protocols approved by the Second Qilu Hospital of Shandong University. Peripheral blood mononuclear cells were isolated by density gradient centrifugation over Ficoll–Paque and washed with PBS without Ca^2+^/Mg^2+^. T-cells were isolated using Dynabeads FlowComp Human CD3 (Thermo Fisher Scientific) according to the manufacturer’s protocol.

Fresh T-cells were cultured in complete X-VIVO medium containing 100 U/mL recombinant human interleukin-2 (rIL-2, Novoprotein) and CD3/CD28 Dynabeads (Thermo Fisher Scientific). One day after stimulation with beads, the cells were transduced with the desired lentivirus at an multiplicity of infection (MOI) of 5. The cells were cultured in complete medium supplemented with cytokines, and the medium was refreshed every 2 days. On day 7, the magnetic beads were removed and the cells were further cultured until approximately day 11.

### Murine T cell isolation, transduction, and expansion

Murine T cells were isolated from the spleens of C57BL/6 mice using a CD3 T Cell Isolation Kit (BioLegend). Purified T cells were cultured in RPMI 1640 medium supplemented with 10% fetal bovine serum, 1% penicillin–streptomycin, 10 mM HEPES, 1 mM sodium pyruvate, 55 nM β-mercaptoethanol, and 100 U/mL recombinant mouse IL-2 (mIL-2, Novoprotein). T cells were activated using CD3/CD28 Dynabeads (Gibco) for 24 hours. Subsequently, cells were transduced with retroviral supernatant in the presence of Vectofusin (Miltenyi Biotec) by centrifugation at 400 × g for 1 hour at room temperature. Cells were cultured for an additional 7 days, after which magnetic beads were removed prior to use in both *in vitro* and *in vivo* assays.

### Stable cell lines

Stable expression of SpyCatcher/SpyTag or luciferase in the target cells was achieved using lentiviral transduction. Target cells were seeded into 6-well plates, and lentivirus was added 24 h later. After 24 h of infection, the medium was replaced with complete culture medium. At 3 days post-infection, the cells were passaged and subjected to selection using puromycin or hygromycin for 1 week. Stable expression was confirmed by flow cytometry and/or fluorescence imaging before subsequent experiments. The transduced cell populations were used for *in vitro* assays and tumor model establishment.

### CAR-T cell-mediated cytotoxicity assay

For the luminescence-based cytotoxicity assay, target cells stably expressing luciferase were used to quantify cell death based on luminescence intensity. A total of 2 × 10^4^ target cells were seeded in 100 μL of target cell culture medium per well in a 96-well plate. CAR-T cells or scramble T cells were added at various effector-to-target (E:T) ratios in 100 μL of X-VIVO medium per well. After 24–48 h of co-culture in a cell incubator, and the luminescence intensity was measured using a luciferase assay kit.


$$\%\text{Cytotoxicity}=\frac{\left(\text{Scramble}-\text{treated luminescence intensity}-\text{Target cells luminescence intensity}\right)}{\text{Scramble}-\text{treated luminescence intensity}}\times 100$$

For the flow cytometry-based cytotoxicity assay, T cells were labeled with a cell proliferation staining reagent, deep red fluorescence cytopainter (E_x_/E_m_=628/643; Abcam, ab176736), and co-cultured with SpyTag-labeled target cells at an E:T ratio of 1:1. After 24 h of incubation, the number of unlabeled cells was quantified by flow cytometry. Cytotoxicity was calculated using the following formula:


$$\%\text{Cytotoxicity}=\frac{\left({\text{Scramble-treated APC}}^{-}\text{ cell number}-{\text{APC}}^{-}\text{ cell number}\right)}{{\text{Scramble-treated APC}}^{-}\text{ cell number}}\times 100$$

### CAR-T cell proliferation

T cells were labeled with the cytoplasmic cell proliferation staining reagent, according to the manufacturer’s protocol, and co-cultured with SpyTag-labeled target cells. After incubation, the T cells were analyzed using flow cytometry.

### Intracellular/extracellular staining and flow cytometry

For flow cytometry analysis, the cells were resuspended in FACS buffer [PBS without Ca^2+^ and Mg^2+^, containing 1% bovine serum albumin (BSA)]. For detecting MYC, cells were incubated with primary antibody (Myc-Tag (9B11) Mouse mAb, Cell Signaling Technology, #2276) for 30 minutes at 4 °C. After washing, cells were incubated with a second antibody (ABclonal, AS139) for 30 min at 4 °C. For extracellular staining, the cells were stained for CD69, CD4, CD8, CD45, CD62L, PD-1 (programmed death-1), TIM-3 (T-cell immunoglobulin and mucin domain 3), and LAG-3 (lymphocyte-activation gene 3). For cytokine and intracellular staining, the cells were incubated with brefeldin A (BioLegend # 420601) for 12 h, fixed (BioLegend, #420801), permeabilized (BioLegend, #421002), and processed according to the manufacturer’s instructions. Cells were then incubated antibodies for 30 min at 4 °C in the dark and washed three times prior to resuspension in FACS buffer and acquisition on the NovoCyte D2040R (Agilent). Data were analyzed using the FlowJo software (v10, TreeStar).

### Enzyme-linked immunosorbent assay

CAR T cells (1 × 10^4^ in 100 μL X-VIVO without cytokine) were added to 96 wells. Where specified, target/off-target cells (1 × 10^4^ in 100 μL X-VIVO without cytokine) were incubated with T cells wells (final volume of 200 μL). Supernatants were collected at various incubation time by centrifugation and stored at −80 °C until used. ELISA was processed according to the Human IFNγ ELISA Kit (DAKEWE, 1110002) manufacturer’s protocol. The plates were read at 450 nm using a SuperMax3100 Counter (Shanghai Flash Spectrum Biochem Technologies Co. Ltd.).

### Nuclear Factor of Activated T-cells reporter

The Nuclear Factor of Activated T-cells (NFAT) reporter construct consisted of four NFAT-response elements (NFAT-REs) and an IL-2 minimal promoter driving luciferase expression, and included a puromycin resistance gene. The NFAT construct was stably introduced into Jurkat cells via lentiviral transduction. The CAR constructs were then delivered into NFAT-Jurkat cells by lentiviral transduction. At 3 days post-transduction, NFAT-CAR-Jurkat cells were co-cultured with SpyTag-positive or SpyTag-negative cells in a 1:1 ratio for 24 h and luciferase activity was measured using a SuperMax3100 Counter.

### Cellular binding assay of SpyCatcher CAR and SpyTag

SpyTag-positive cells were stained with a cytopainter (E_x_/E_m_=628/643, Abcam, ab176736) according to the manufacturer’s protocol and co-cultured with CAR-Jurkat cells expressing SpyCatcher CAR-GFP at an E:T ratio of 1:5 in a glass-bottomed cell culture dish (NEST, 801021) for 12 h using a high-speed confocal microscopy system (Dragonfly 200).

### *In vivo* tumor studies

All animal experiments were performed using protocols approved by Scientific Research Ethics Committee Member of the Second Qilu Hospital of Shandong University (approval no. KYLL202602076). All animal euthanasia procedures were performed in accordance with the AVMA Guidelines for the Euthanasia of Animals: 2020 Edition. Mice were euthanized by gradual CO_2_ inhalation using a displacement rate of 30% of the chamber volume per minute. After respiratory arrest, CO_2_ exposure was maintained for at least 1 min, and death was confirmed by cervical dislocation. For procedures requiring anesthesia, mice were anesthetized with isoflurane (3% for induction and 1.5–2% for maintenance in oxygen). The number of animals per group is indicated in each figure legend. Sample sizes were determined based on previous experience with similar tumor models and published studies, considering ethical reduction of animal use. Cells were transduced with a lentivirus carrying firefly luciferase for non-invasive optical imaging.

For subcutaneous tumor studies, PC3 cells or PC3 stable expressing SpyTag (PC3-ST) (1×10^6^) were prepared in PBS and injected subcutaneously into the abdomen of male NSG mice on day 0. Mice were randomly assigned into experimental groups (n = 5 per group). On days 10 and 20, mice received tail vein injections of 1×10^6^ SpyCatcher CAR-T cells or Scramble T cells. Tumor size was measured every 10 days using calipers, and tumor volume was calculated using the formula: *V*=1/2×length×width^2^. Bioluminescence imaging was performed on days 30, 40, and 50. Mice were sacrificed on day 50.

For OV combined with CAR-T therapy in PC3 xenograft model, PC3 cells (1×10^6^) were subcutaneously injected in the abdomen of male mice on day 0. On day 35, mice with comparable tumor volumes were selected and randomly allocated into treatment groups (n = 5 per group) to minimize baseline differences among groups. Mice received an intratumoral injection of 1×10^6^ plaque-forming units (PFU) of OV in 100 μL PBS. On day 40, 5 × 10^6^ CAR-T cells or Scramble T cells were administered via tail vein injection. On day 60, the mice were euthanized and tumors were harvested for flow cytometric analysis or immunohistochemistry, as described below.

For immunocompetent syngeneic tumor studies, male C57BL/6 mice were subcutaneously inoculated with 5 × 10^6^ TRAMP-C1 cells in the abdomen on day 0. When tumors reached comparable sizes, mice were randomly assigned into indicated treatment groups (n = 10 per group). On day 25, mice received intratumoral injection of 1 × 10^6^ PFU of OAD-SpyTag or control treatment in 100 μL PBS. On day 28, mice were administered 5 × 10^6^ murine SpyCatcher CAR-T cells or mock T cells via intratumoral injection. Tumor growth was monitored every 3 days using calipers.

### Immunohistochemistry and hematoxylin–eosin

The tumors were fixed in paraformaldehyde for 2 days and embedded in paraffin. Paraffin-embedded sections (4u*m) were subjected to hematoxylin and eosin (H&E) staining according to manufactory’s protocol (Solarbio, 2500020002). For immunohistochemical, sections* were stained with MYC (Cell Signaling Technology, #2276), CD3 (Abcam, ab16669), Hexon (Abcam, ab316852) using immunohistochemistry (ich) kit (ZSGB-BIO, PV6000) following the manufacturers’ protocols. Images were obtained using the Nikon 90i.

### Oncolytic adenovirus packaging, purification, and titer determination

The pShutter-SpyTag plasmid was constructed by inserting the human telomerase reverse transcriptase (hTERT) promoter–E1A–E1B19K–transferrin receptor transmembrane domain–Ag85B–MYC–SpyTag (–P2A–sfGFP) cassette into the AgeI/SpeI sites of pShutter. After linearization with PmeI, the plasmid was transformed into BJ5183-pAdEasy-1 cells to generate the recombinant plasmid, pAdEasy-SpyTag. The sequence was verified using Sanger sequencing. pAdEasy-SpyTag was linearized with PacI and transfected into 293A cells using PEI for adenovirus production. After sufficient cytopathic effects, the cells were harvested and subjected to repeated freeze–thaw cycles to obtain the first-generation (F1) oncolytic adenovirus. The virus was amplified at an infection ratio of 1:10.

The crude adenovirus was purified using Capto Q ImpRes affinity chromatography (Cytiva, 17547003). The viral supernatant was buffer-exchanged with binding buffer (20 mM Tris, 300 mM NaCl, pH 8.0), followed by column binding, washing with washing buffer (20 mM Tris, 480 mM NaCl, pH 8.0), and elution with elution buffer (20 mM Tris, 700 mM NaCl, pH 8.0). Eluted virus was collected and buffer-exchanged into PBS using 50 kDa centrifugal filter (Millipore, UFC905024), then stored at −80 °C and used as soon as possible.

A rapid detection kit (Sangon, B605105) was used to determine the adenoviral titer. 293A cells (2.5×10^5^/well) were seeded and infected with serial dilutions of adenovirus after 1 h. After 48 h of incubation, cells were harvested and fixed with methanol. After three washes, cells were blocked with 1% BSA at 37 °C for 1 h and stained with anti-Hexon antibody for 1 h at 37 °C. After washing, the cells were incubated with fluorescent secondary antibodies and the percentage of positive cells was determined by flow cytometry.

### Cytotoxicity mediated by oncolytic adenovirus *in vitro*

PC3-luc cells (1×10^5^/well) were seeded into 24-well plates. After cell adherence, oncolytic adenoviruses were added at different MOIs. The cells were incubated for 24, 48, or 72 h. At each time point, all the cells were harvested for analysis. Expression of SpyTag-sfGFP and the hexon of the adenovirus was evaluated by flow cytometry, and cell death was assessed by measuring luciferase as previously described.

### Combined cytotoxicity of OV and CAR-t cells *in vitro*

Target cells stably expressing the luciferase were seeded and allowed to adhere. Oncolytic adenoviruses were added at different MOIs. After 6 h, SpyCatcher CAR-T or scrambled T cells were added at various E:T ratios. The co-cultures were incubated for various durations. Cytotoxicity was assessed by measuring luciferase activity, as previously described.

### Prostate cancer organoid establishment

Organoid establishment was performed following protocols with modifications (38, 39). Briefly, tumor specimens were washed in ice-cold Advanced Dulbecco’s Modified Eagle Medium/F12 (DMEM/F12), minced into 0.1~0.5 mm³ fragments, and digested in 5 mg/ml Collagenase Type II at 37 °C for 2 h with intermittent trituration, followed by dissociation in TrypLE Express supplemented with 10 μM Y-27632 at 37 °C for 10 min. DNase I (50 μg/ml) was added to reduce DNA viscosity, and the cell suspension was filtered through a 70 μm strainer. After centrifugation (300 × g, 5 min), cells were counted and resuspended in 90% Matrigel at 20,000 cells per 40 μl. Droplets (40 μl) were seeded in pre-warmed 24-well plates, solidified by inversion at 37 °C for 30 min, and overlaid with 500 μl complete organoid culture medium. Cultures were maintained at 37 °C with 5% CO_2_, and medium was refreshed every 3–4 days. Organoids were embedded in optimal cutting temperature compound (OCT), and used for H&E and immunofluorescence staining.

The basal medium consisted of Advanced DMEM/F12 supplemented with 1× GlutaMAX (Gibco, 35050-038), 10 mM 4-(2-hydroxyethyl)-1-piperazineethanesulfonic acid (HEPES) (Gibco, 15630-056), and 1× penicillin-streptomycin. The complete organoid culture medium was prepared by supplementing the basal medium with the following growth factors and small molecules: 500 ng/ml recombinant human R-spondin 1 (R&D Systems, 3474-RS), 100 ng/ml recombinant human Noggin (MedChemExpress, HY-P70558), 20 ng/ml recombinant human Wnt3a (R&D Systems, 5036-WN), 50 ng/ml recombinant human Hepatocyte Growth Factor (HGF; MedChemExpress, HY-P704662), 10 ng/ml recombinant human FGF10 (R&D Systems, 345-FG), 50 ng/ml recombinant human Epidermal Growth Factor (EGF; R&D Systems, 236-EG), 5 ng/ml recombinant human FGF2/bFGF (R&D Systems, 233-FB), 1× N21-MAX Media Supplement (R&D Systems, AR008), 10 mM Nicotinamide (Sigma, N0636), 1.25 mM N-acetyl-L-cysteine (NAC; Sigma, A9165), 10 μM SB 202190 (Sigma, S7067), 500 nM A83-01 (Tocris, 2939), 1 μM Prostaglandin E2 (PGE2; Sigma, P5640), and 10 nM Dihydrotestosterone (DHT; Sigma, D073). For the first week after passaging or thawing, 10 μM Y-27632 dihydrochloride (Sigma, Y0503) was additionally included.

### OAD infection and CAR-T cell-mediated killing assay in patient-derived prostate cancer organoids

To evaluate OAD-mediated antigen delivery and CAR-T cell-mediated cytotoxicity in patient-derived prostate cancer organoids, organoids of uniform size (approximately 100–200 μm in diameter) were manually selected and transferred. Selected organoids were stained with Hoechst 33342 (1 μg/ml) for 15 min at 37 °C to label nuclei, and transferred to a 96-well plate in X-VIVO medium containing 0.5 μg/ml propidium iodide to monitor cell death. OAD was added at a MOI = 1 based on the estimated cell number per organoid. At 6 h post-infection, 2 × 10^4^ CAR-T cells or scramble T cells, pre-labeled with 1 μM carboxyfluorescein succinimidyl ester (CFSE; Invitrogen, C34554), were added to each well. Organoid–T cell co-cultures were incubated for an additional 24 h at 37 °C with 5% CO_2_. At the endpoint, plates were imaged on an inverted fluorescence microscope using Z-stack acquisition to capture the full three-dimensional volume of each organoid. Images were acquired across three channels: Hoechst 33342 (organoid nuclei), CFSE (T cells), and PI (dead cells). T cell binding was quantified as the ratio of CFSE-positive area to Hoechst 33342-positive area per organoid. Organoid killing was quantified as the ratio of PI-positive area to Hoechst 33342-positive area per organoid. Image analysis was performed using ImageJ software (NIH, v 2.16.0). At least three independent experiments were performed.

### Statistical analysis

Data are presented as means ± standard errors of the mean, unless otherwise stated. Groups were compared using an unpaired two-tailed Student’s t-test to calculate the P-value unless otherwise stated. Significance was set as follows: *p < 0.05, **p < 0.01, ***p < 0.001, and ns indicated no significance.

## Results

### SpyCatcher formed mechanically stable covalent bond with SpyTag

**Figure 1:**
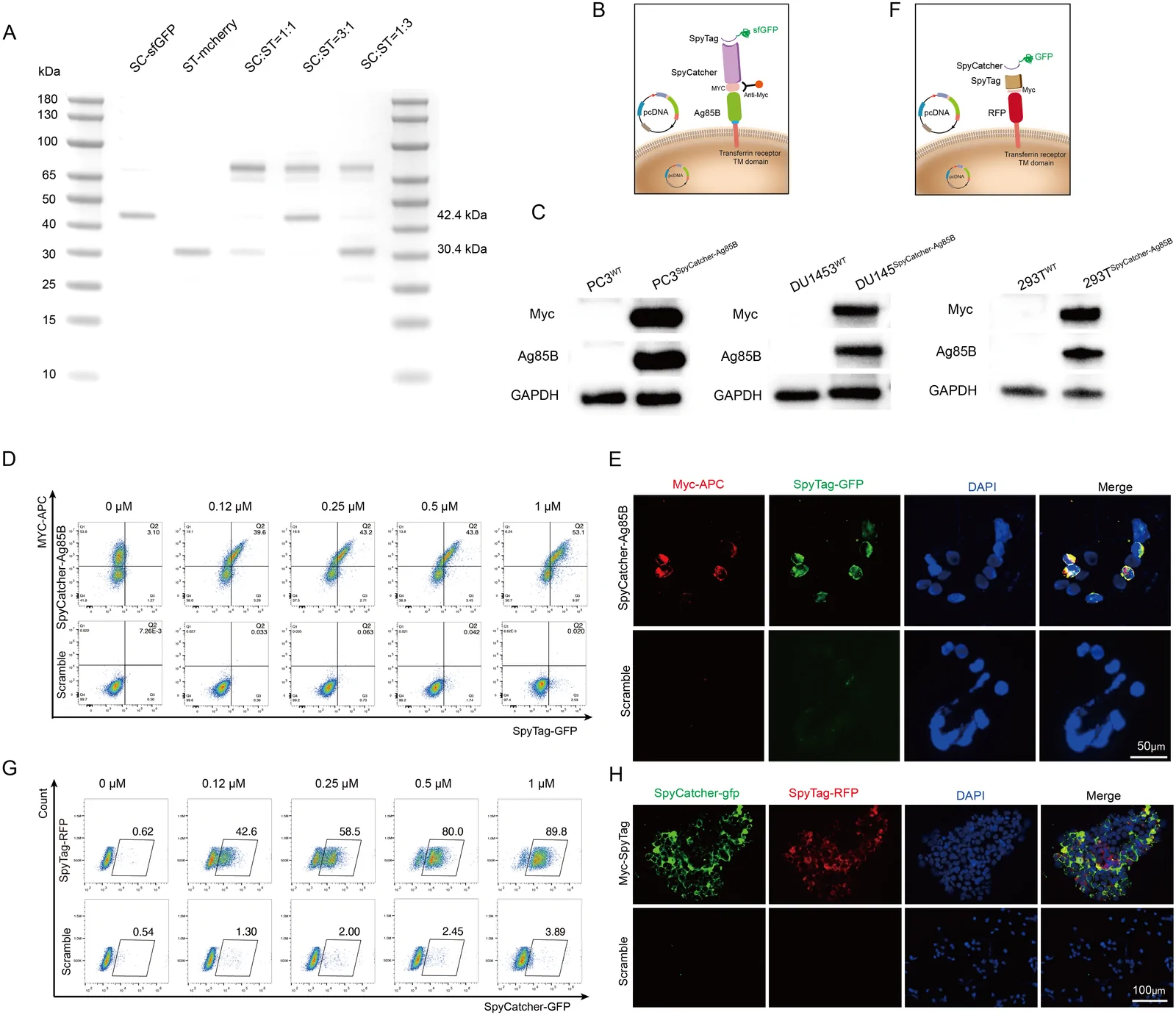
SpyCatcher formed mechanically stable covalent bond with SpyTag. **(A)** SDS-PAGE gel electrophoresis with Coomassie blue staining. Lanes from left to right: Marker; SpyCatcher-sfGFP (SC-sfGFP, 42.4 kDa); SpyTag-mCherry (ST-mCherry, 30.4 kDa); SpyCatcher-sfGFP and SpyTag-mCherry mixed at a 1:1 mass ratio and incubated at room temperature for 10 min; SpyCatcher-sfGFP and SpyTag-mCherry mixed at a 3:1 mass ratio and incubated at room temperature for 10 min; SpyCatcher-sfGFP and SpyTag-mCherry mixed at a 1:3 mass ratio and incubated at room temperature for 10 min; marker. **(B, C)** Schematic representation of the Ag85B-SpyCatcher expression vector (Transferrin Receptor cytosolic region and transmembrane helix [TfR], Ag85B, Myc-tag, SpyCatcher) and western blotting to confirm protein expression in PC3, DU145, and 293T cells. **(D)** Flow cytometric analysis of SpyTag-sfGFP binding to Ag85B-SpyCatcher-expressing cells. SpyTag-sfGFP was expressed and purified from *Escherichia coli*. **(E)** Confocal microscopy showing the colocalization of SpyCatcher (APC-anti-Myc, red) and SpyTag-sfGFP (green). **(F)** Schematic representation of SpyTag expression vector (TfR, RFP, Myc-tag, and SpyTag). **(G)** SpyCatcher-sfGFP was expressed and purified from *E. coli* bound to SpyTag-displaying cells. The sfGFP^+^ cells were quantified using flow cytometry. **(H)** Fluorescence imaging of the assembled complexes.

We expressed and purified the fusion proteins SpyCatcher-sfGFP (42.4 kDa), SpyTag-mCherry (30.4 kDa), SpyCatcher-mCherry (42.3 kDa), and SpyTag-sfGFP (30.7 kDa) using a prokaryotic expression system. SpyCatcher-sfGFP and SpyTag-mCherry were mixed at varying mass ratios, incubated for 10 min at room temperature, and separated by SDS-PAGE (**Figure 1A**, **Supplementary Figure 1**). The SpyCatcher and SpyTag formed covalent fusion proteins that remained stable and resistant to denaturation at 95 °C in denaturation buffer.

We developed an antigen display platform by engineering a chimeric protein (Ag85B-SpyCatcher) that integrates the *M. tuberculosis* antigen Ag85B, Myc-tag, and SpyCatcher domains. The construct was anchored to the plasma membrane via the transmembrane and cytosolic domains of human transferrin receptor (TfR) (**Figure 1B**). Western blot analysis confirmed successful expression of the full-length Ag85B-SpyCatcher fusion protein (~55 kDa) in PC3, DU145, and 293T cells (**Figure 1C**). Functional validation of the SpyCatcher/SpyTag interaction was performed by incubating SpyCatcher-expressing cells with bacterially produced SpyTag-sfGFP. Flow cytometry demonstrated a concentration-dependent increase in dual MYC/sfGFP-positive cells (**Figure 1D**). The specificity of the interaction was confirmed by the absence of the sfGFP signal in the untransfected cells. Confocal microscopy further revealed the strong co-localization of SpyCatcher and SpyTag-sfGFP on the cell surface (**Figure 1E**). To test the bidirectional compatibility of the system, we displayed SpyTag on cell surfaces and exposed them to SpyCatcher-sfGFP (**Figure 1F**). Flow cytometry quantified a concentration-dependent sfGFP signal, whereas fluorescence imaging confirmed the uniform membrane localization of the complexes without intracellular aggregation (**Figures 1G, H**).

Collectively, these results establish the SpyCatcher/SpyTag system as a rapid, high-affinity tool for programmable surface engineering in mammalian cells, with potential applications in antigen presentation and synthetic biology.

### SpyCatcher CAR-T selectively targeted and lysed SpyTag-expressing cells

**Figure 2:**
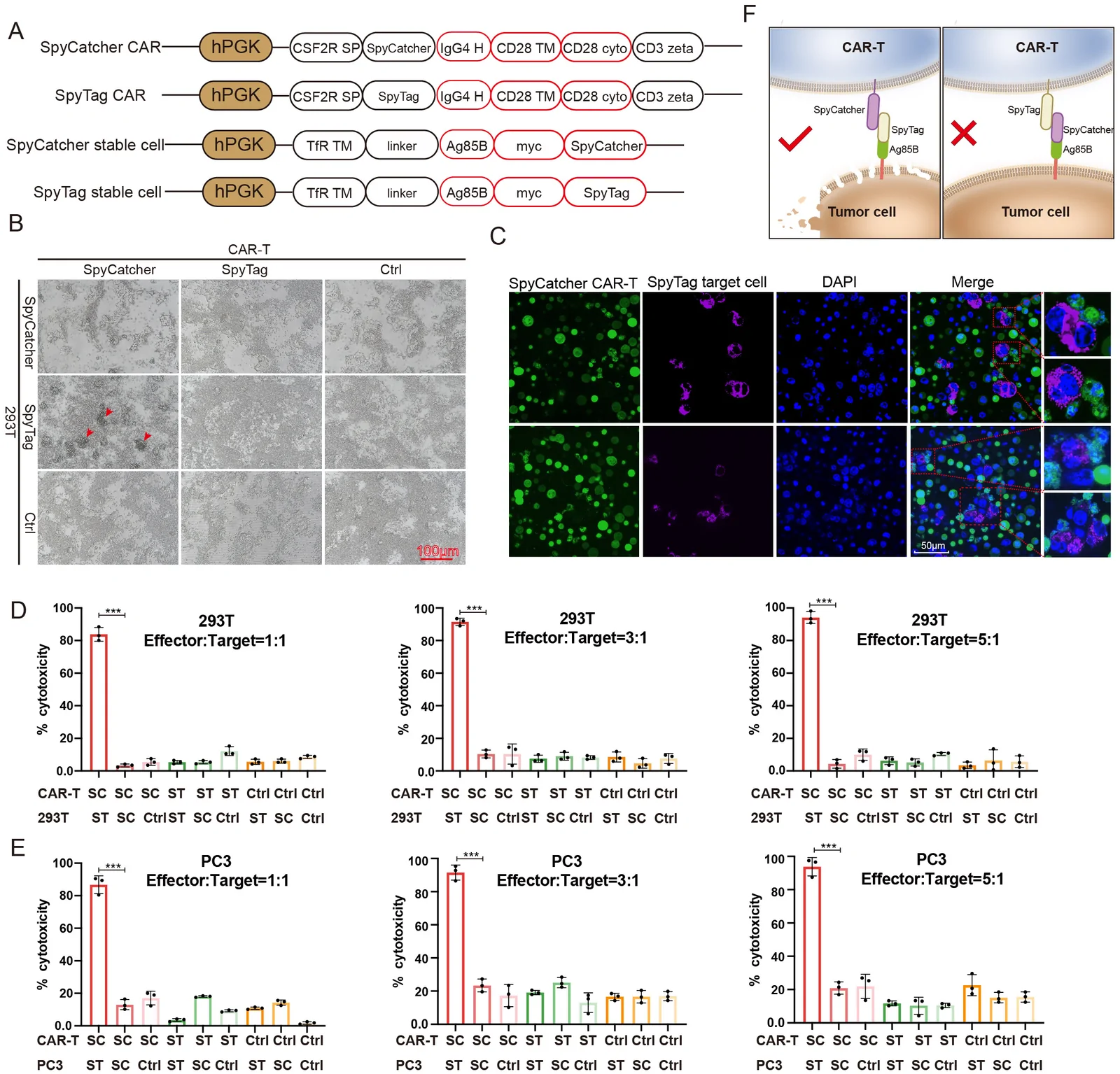
SpyCatcher CAR-T selectively targeted and lysed SpyTag-expressing cells. **(A)** Schematic designs of SpyCatcher CAR (hPGK promoter-CSF2R-SpyCatcher-IgG_4_ hinge-CD28-CD3ζ) and SpyTag CAR constructs, along with SpyCatcher/SpyTag-engineered target cells. **(B)** Bright-field microscopy of co-cultures between SpyCatcher CAR-T, SpyTag CAR-T, or control CAR-T and 293T cells expressing SpyCatcher, SpyTag, or empty vector (Ctrl). Red arrows indicate large aggregated cell clusters formed exclusively in SpyCatcher CAR-T/SpyTag-293T groups. **(C)** Confocal imaging of SpyCatcher CAR-T (green) and CellTrace-labeled SpyTag-293T cells (red), showing direct cell–cell contact. **(D)** Cytotoxicity of SpyCatcher CAR-T against SpyTag-293T cells at E:T ratios of 1:1, 3:1, and 5:1. Luciferase activity was measured after a 12-hour co-culture. **(E)** SpyCatcher CAR-T-mediated lysis of SpyTag-PC3 prostate cancer cells at varying E:T ratios. Data normalized to untreated controls. **(F)** Model of unidirectional specificity: SpyCatcher CAR-T activation requires SpyTag on target cells, whereas SpyTag CAR-T fails to engage SpyCatcher-expressing targets. ***, p < 0.001.

To explore the SpyCatcher/SpyTag system as a covalent linkage platform for CAR-T therapy, we engineered two CARs: (1) SpyCatcher CAR (hPGK promoter-CSF2R signal peptide-SpyCatcher-IgG_4_ hinge-CD28-CD3ζ) and (2) SpyTag CAR (hPGK promoter-CSF2R signal peptide-SpyTag-IgG_4_ hinge-CD28-CD3ζ). Both constructs were stably transduced into T-cells via lentiviral delivery. In parallel, SpyCatcher- or SpyTag-expressing 293T and PC3 cells were generated as target models (**Figure 2A**). Coculture of SpyCatcher CAR-T cells with SpyTag-expressing 293T cells induced rapid cell clustering within 12 h, with large aggregates visible under a bright-field microscope (**Figure 2B**). By contrast, the control groups (SpyTag CAR-T + SpyCatcher-293T and Ctrl CAR-T + SpyTag-293T) showed no aggregation, indicating strict dependency on SpyCatcher/SpyTag pairing (**Figure 2B**). Confocal imaging of CFSE-labeled SpyTag-293T cells (red) and GFP^+^ SpyCatcher CAR-T cells (green) confirmed direct membrane contact at the interface of the conjugated cells (**Figure 2C**). The functional specificity was quantified using a luciferase-based cytotoxicity assay. At a 1:1 effector-to-target (E:T) ratio, SpyCatcher CAR-T cells lysed greater than 85% of SpyTag-293T cells within 12 h, compared to <5% killing in the non-paired groups (**Figure 2D**). The cytolytic activity was dose-dependent, increasing to 90%~96% at E:T ratios of 3:1 and 5:1, respectively (**Figure 2D**). A similar efficacy was observed against SpyTag-expressing PC3 prostate cancer cells (**Figure 2E**). These results established SpyCatcher/SpyTag as a programmable CAR-T platform, where covalent SpyCatcher CAR/SpyTag target pairing is essential for triggering T cell activation and tumor-specific killing (**Figure 2F**).

### Structural optimization of SpyCatcher CAR-T exhibited potent cytotoxicity *in vitro* and *in vivo*

**Figure 3:**
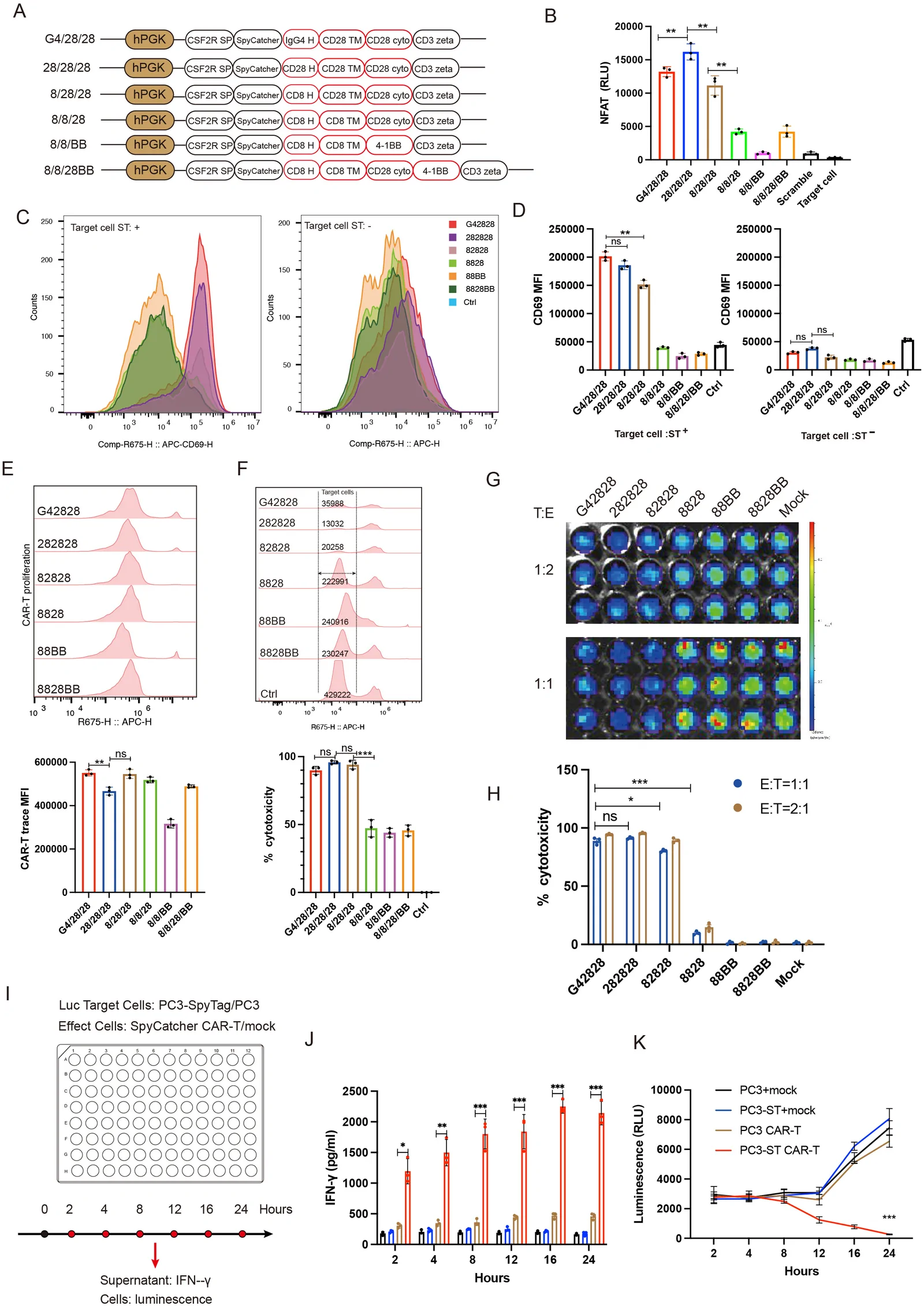
Structural optimization of SpyCatcher CAR-T exhibited potent cytotoxicity *in vitro.* **(A)** Schematic of six CAR architectures tested, including second- (G4/28/28, 28/28/28, 8/28/28, 8/8/28, 8/8/BB) and third-generation (8/8/28BB) designs. Co-stimulatory domains (CD28 or/and 4-1BB) and hinge regions (IgG_4_, CD28, CD8) are annotated. **(B)** NFAT-luciferase reporter cells (luciferase was promoted by IL-2, NFAT-binding domains promoter) were transduced with each CAR construct and co-cultured with SpyTag-expressing target cells. **(C, D)** CD69 expression in six CAR-T cells post-target/off-target engagement. Representative flow cytometry plots in **(C)**, quantification in **(D, E)** Proliferation of human CAR-T cells (day 9 post-lentiviral transduction) assessed by CellTrace dilution after 48 h of co-culture with target cells. **(F)** Cytotoxic activity against SpyTag^+^ targets. Target cell depletion quantified by flow cytometry after 24-hour co-culture with CAR-T cells. Target cells labeled with CellTrace and CAR-T expressed GFP tag. **(G, H)** Luciferase-based killing assay measuring residual luminescence in surviving targets. **(I)** Schematic of CAR-T killing kinetics. Target cells consisted of luciferase-expressing PC3-SpyTag or wild-type PC3 cells, while effector cells were either SpyCatcher CAR-T or mock T cells (mock). These were co-cultured at a 1:1 ratio. Supernatants were collected at 2, 4, 8, 12, 16, and 24 hours to measure IFN-γ levels **(J)**, and luciferase activity was monitored to assess target cell viability **(K)**. Data are presented as means ± standard deviations; *p < 0.05, **p < 0.01, ***p < 0.001.

To improve the SpyCatcher CAR-T cell efficacy, we engineered six CAR architectures (second- and third-generation) that incorporated distinct co-stimulatory domain combinations (CD28 and/or 4-1BB) and hinge regions (IgG_4_, CD28, or CD8) (**Figure 3A**). CAR activity was systematically evaluated using an NFAT-luciferase reporter system in Jurkat cells, which stably express IL-2, NFAT-binding domains, and luciferase. Upon co-culture with SpyTag-expressing targets, the 28/28/28 CAR demonstrated superior cytotoxicity, eliciting a two-fold higher luciferase activity than 8/8/28 CARs and minimal activity in 8/8/BB and scramble (**Figure 3B**). Target cells robustly activated 28/28/28 CAR-T cells, as evidenced by the 5-fold upregulation of CD69 versus 8/8/BB CAR-T cells (**Figures 3C, D**; **Supplementary Figure 2**).

**Figure 4:**
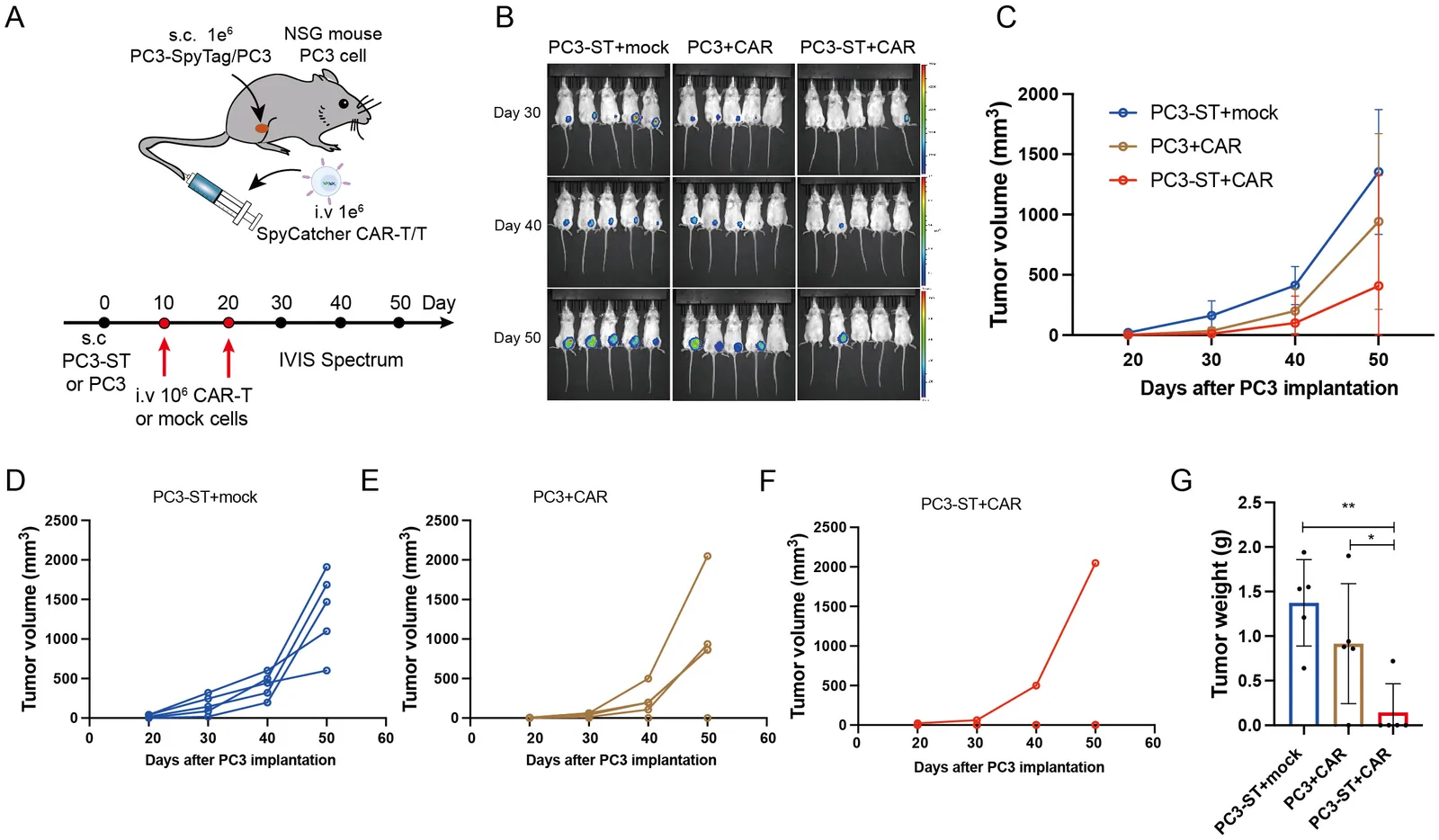
SpyCatcher CAR-T exhibited potent cytotoxicity *in vivo*. **(A)** Schematic of tumor-killing by SpyCatcher CAR-T cells in NSG mouse (n=5/group). On day 0, mice were subcutaneously inoculated with 1×10^6^ PC3-SpyTag (PC3-ST) or wild-type PC3 cells. SpyCatcher CAR-T cells or mock T cells were administered via tail vein injection on days 10 and 20. PC3-ST + mock: inoculated with PC3-SpyTag cells and treated with mock T cells; PC3 + CAR: inoculated with wild-type PC3 cells and treated with SpyCatcher CAR-T cells; PC3-ST + CAR: inoculated with PC3-SpyTag cells and treated with SpyCatcher CAR-T cells. **(B)** Tumor growth was monitored by *in vivo* luciferase imaging on days 30, 40, and 50. **(C–F)** Tumor volume growth curves. **(G)** Tumor weight measured at the end of the experiment. *, p < 0.05; **, p < 0.01.

Primary human T-cells transduced with SpyCatcher CAR variants exhibited distinct functional profiles. Although 8/8/BB CAR-T cells exhibited greater CellTrace dilution after 48 h of co-culture with target cells compared to other groups, consistent with increased proliferation, this construct did not show detectable cytotoxicity in prior assays (**Figure 3E**). Co-culture of GFP-expression SpyCatcher CAR-T cells with CellTrace SpyTag^+^ targets revealed potent antigen-specific killing by 28/28/28, G4/28/28, and 8/28/28 CAR-T cells, eliminating >90% of the target cells within 24 h, whereas 8/8/28, 8/8/BB, and 8/8/28BB variants showed negligible activity (<50%) (**Figure 3F**; **Supplementary Figure 3**). Following antigen exposure, 28/28/28 CAR-T cells displayed relatively low expression of exhaustion-associated markers PD-1, TIM-3, and LAG-3 (**Supplementary Figures 4A, B**). Furthermore, after co-culturing different CAR-T cells with target cells expressing luciferase for 24 hours, cytotoxicity was assessed using IVIS luminescence imaging. Both the 28/28/28, G4/28/28, and 82828 CAR-T variants demonstrated significant killing activity (**Figures 3G, H**). Kinetic analyses demonstrated progressive accumulation of IFN-γ over 24 h, with target lysis becoming statistically significant by 12 h (**Figures 3I–K**). To evaluate the antitumor efficacy of SpyCatcher CAR-T cells *in vivo*, PC3 cells stably expressing SpyTag (PC3-ST) or wild-type PC3 cells were subcutaneously inoculated into mice on day 0. SpyCatcher CAR-T cells or mock T cells were administered via tail vein injection on days 10 and 20 (**Figure 4A**). Treatment with SpyCatcher CAR-T cells significantly suppressed tumor growth in mice bearing PC3-ST tumors (**Figures 4B–G**). These data conclusively established 28/28/28 SpyCatcher CAR-T cells as potent therapeutic candidates with balanced proliferation, cytotoxicity, and durability.

### Synergistic cytotoxic effects of OAD-SpyTag-Ag85B and SpyCatcher CAR-T *in vitro*

**Figure 5:**
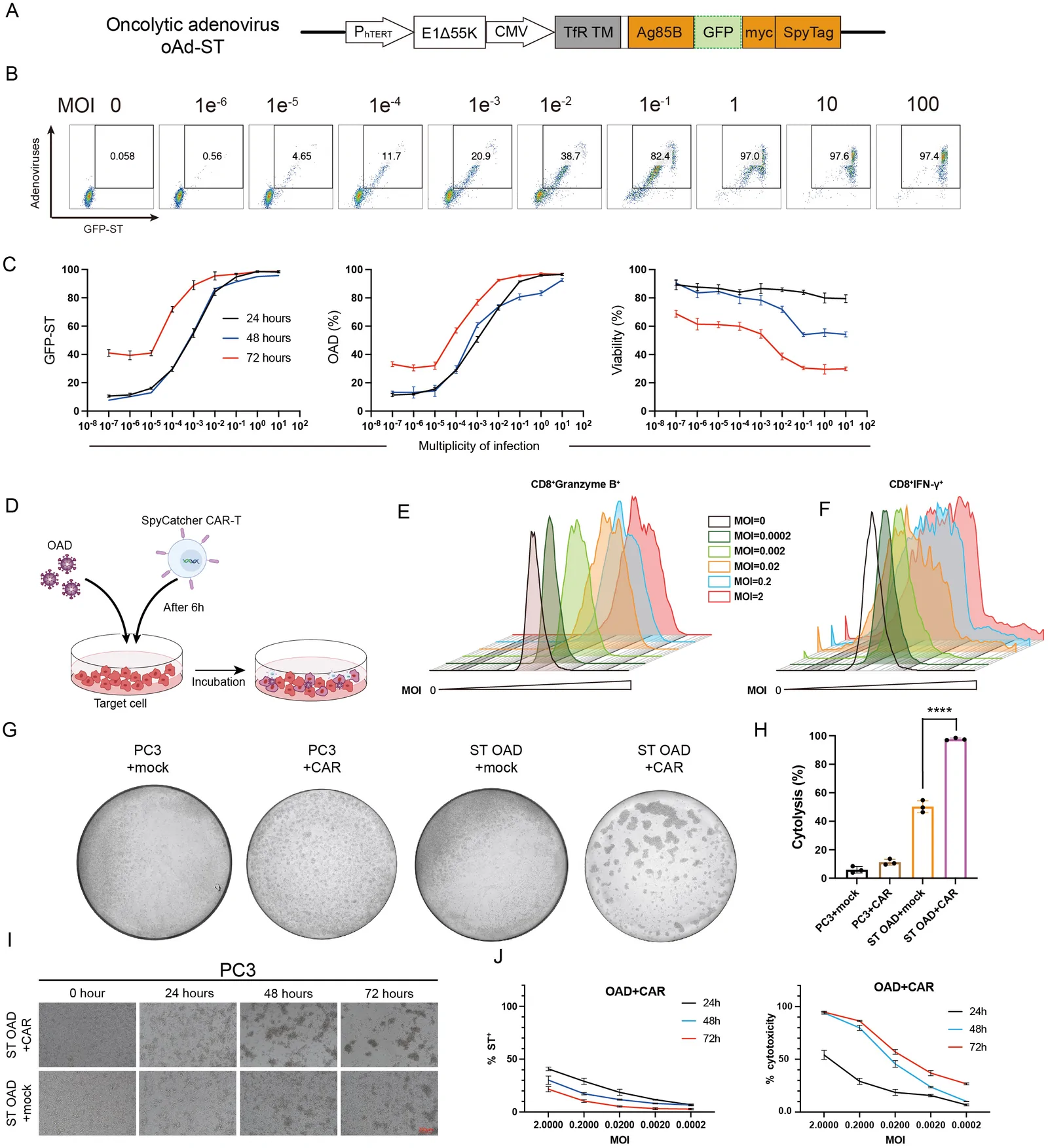
Synergistic cytotoxic effects of Ad-SpyTag-Ag85B and SpyCatcher CAR-T *in vitro*. **(A)** Schematic of the oncolytic adenovirus design. The E1-55K-deleted E1 gene, driven by the human telomerase reverse transcriptase promoter, enables tumor-selective replication in p53-deficient cancer cells. A GFP Tag fusion cassette was incorporated for tracking and removed when unnecessary. **(B)** Dose-dependent transduction efficiency in PC3 cells 24 h post-infection, assessed by GFP expression (green) and adenoviral hexon protein detection. **(C)** Time-course analysis of SpyTag delivery and adenoviral replication. GFP-SpyTag^+^ cell populations (left) and hexon^+^ viral rates (medium) at 24, 48, and 72 h post-infection (MOI = 10^-7^~10). Oncolytic efficacy quantified by cell viability reduction over time (right). **(D)** Schematic diagram of the synergistic cytotoxic effects of OAD and SpyCatcher CAR-T. OAD was added to target cells, and CAR-T cells were added 6 hours later for co-incubation. Protein expression was detected by flow cytometry, and target cell activity was indicated by luciferase. **(E, F)** Flow cytometric analysis of CD8^+^ T cells expressing Granzyme B **(E)** and IFN-γ **(F)** following co-culture with PC3 cells infected with OAD at different multiplicities of infection (MOI). **(G)** Representative bright-field microscopy images of PC3 cells treated with OAD alone or in combination with SpyCatcher CAR-T cells or mock T cells. PC3 cells were first infected with OAD at an MOI of 2. After 6 h, the T cells were added and co-cultured for 24 h. The entire 24-well plate was scanned under a microscope. **(H)** Cytotoxicity was quantified at 24 h using a luciferase-based assay. **(I)** Time-course microscopy images of PC3 cells treated under different conditions for 0, 24, 48, and 72 h. **(J)** Flow cytometric analysis of target protein expression in PC3 cells at 24, 48, and 72 h post-treatment with increasing Ad MOI (0.0002~2). Luciferase assay measures the MOI-dependent and time-dependent killing efficiency among the different treatment groups. ****, p < 0.001.

To enable tumor-targeted delivery of SpyTag, we engineered a replication-defective adenovirus (Ad) with oncolytic properties by introducing an E1B-55K-deleted E1 gene under the control of the hTERT promoter (**Figure 5A**). Infection of PC3 prostate cancer cells with gradient multiplicities of infection (MOI: 0–100) demonstrated dose-dependent transduction efficiency, as evidenced by GFP expression and Hexon protein detection (**Figure 5B**; **Supplementary Figure 5**). Time-course analyses revealed progressive SpyTag-Ag85B expression, with GFP^+^ and oncolytic adenovirus (OAD^+^) populations increasing from 24 h to 72 h (**Figure 5C**; **Supplementary Figure 6**). Concomitant with viral replication, OAD exhibited potent oncolytic activity, reducing PC3 viability (**Figure 5C**). These data establish OAd-SpyTag-Ag85B as a dual-functional vector capable of tumor-selective SpyTag delivery via hTERT-driven replication and direct oncolysis through dose- and time-dependent cytotoxicity.

To evaluate combinatorial antitumor activity, SpyCatcher CAR-T cells were co-cultured with PC3 cells pre-infected with OAD at an effector-to-target (E: T) ratio of 2:1 (**Figure 5D**). Flow cytometry revealed that OAD infection dose-dependently enhanced SpyCatcher CAR-T activation, as evidenced by elevated frequencies of Granzyme B^+^ CD8^+^ T cells (**Figure 5E**; **Supplementary Figure 7**) and IFN-γ^+^ CD8^+^ T cells (**Figure 5F**; **Supplementary Figure 7**). In the full-area scan image of a 24-well plate, neither mock-treated PC3 cells nor SpyCatcher CAR-T cells alone induced cytotoxicity, with the cells maintaining normal adherent morphology (**Figure 5G**). OAD monotherapy (MOI = 2), exhibited limited oncolysis, whereas the combination of OAD and SpyCatcher CAR-T cells induced rapid target cell detachment and aggregation, achieving >90% cytotoxicity after 48 h (luciferase-based assay, **Figure 5H**).

Time- and dose-dependent synergies were further quantified in PC3 models. Luciferase signal decay correlated with both increasing MOI (MOI = 0.0002~2) and prolonged co-culture duration, with maximal cytotoxicity observed at MOI 2 and 72 h (**Figure 5I**). Flow cytometric analysis further showed that the target protein expression in OAD + CAR-T cells declined over time, with positive cell percentages dropping below 20% by 72 h (**Figure 5J**). Cytotoxicity assays confirmed that the combination therapy induced over 90% target cell death at 48 h, whereas the OAD + mock and OAD groups exhibited less than 60% cytotoxicity (**Figure 5J**).

### Synergistic cytotoxic effects of Ad-SpyTag-Ag85B and SpyCatcher CAR-T *in vivo*

**Figure 6:**
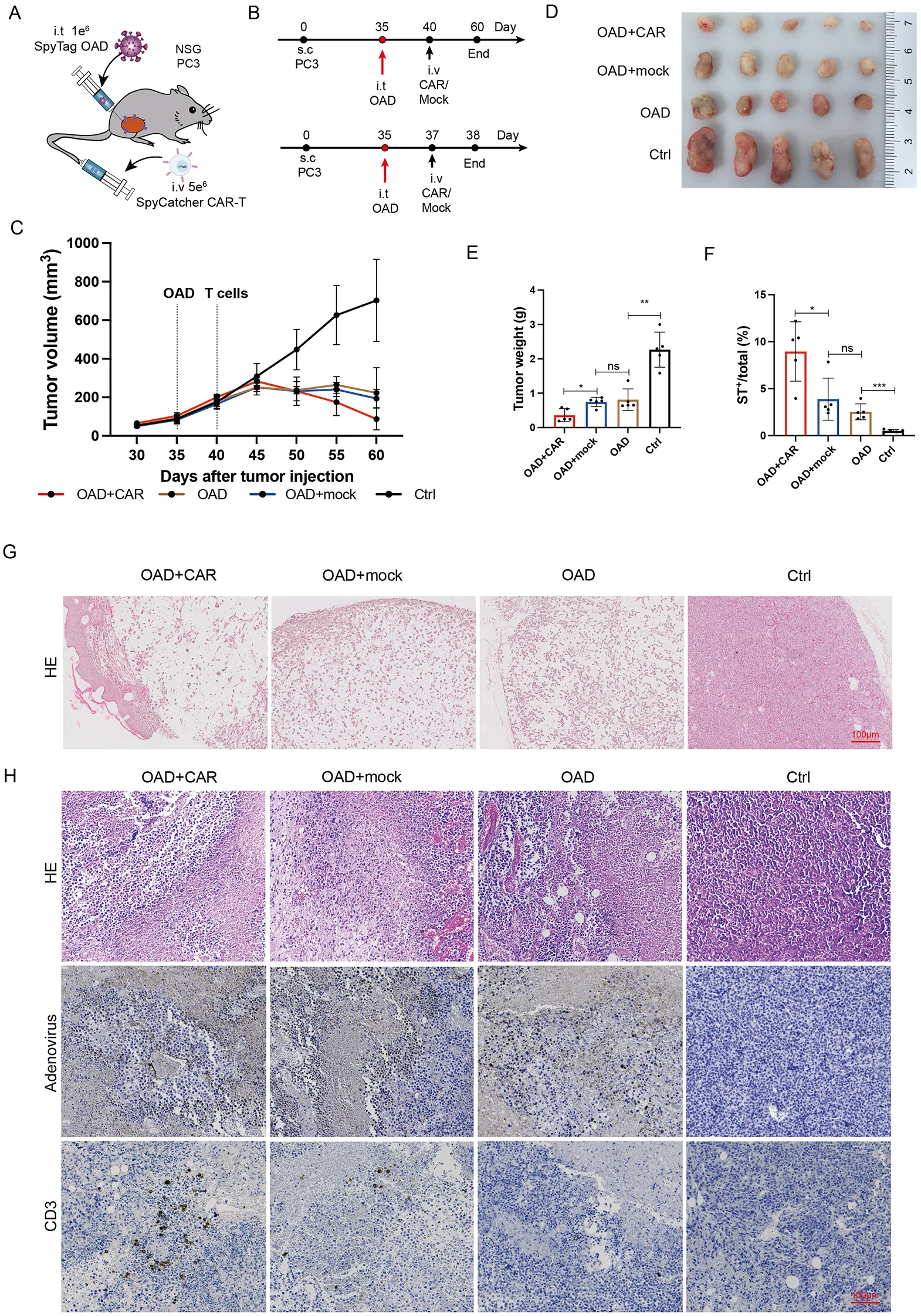
Synergistic cytotoxic effects of Ad-SpyTag-Ag85B and SpyCatcher CAR-T *in vivo*. **(A)** Schematic diagram of the combination treatment with OAD-SpyTag oncolytic adenovirus and SpyCatcher CAR-T cells. Male NOD scid gamma (NSG) mice (n=5/group) were subcutaneously inoculated with 1 × 10^6^ PC3 cells. Intratumoral injection of 1 × 10^6^ PFU OAD and intravenous administration of 5 × 10^6^ SpyCatcher CAR-T cells or mock T cells were performed on days 35 and 40, respectively. **(B)** Schematic of the Experimental Workflow. **(C)** Tumor volume was measured every 5 days, and mice were sacrificed on day 60, and tumor weight was recorded. **(D, E)** Tumor photographs and measurements at the endpoint. **(F)** Single-cell suspensions were prepared from tumor tissues. SpyTag-positive tumor cells were labeled with SpyCatcher-sfGFP. Flow cytometry detected the proportion of positive cells. **(G)** Hematoxylin and eosin staining of tumor tissue. **(H)** HE staining and IHC staining for adenovirus and CD3 were performed on samples from the short-duration experiment (**Figure 6B** lower). *, p < 0.05; **, p < 0.01; ***, p < 0.001.

To evaluate the antitumor activity of the oncolytic adenovirus and SpyCatcher CAR-T cells, NSG mice bearing subcutaneous PC3 tumors were treated with a single intratumoral injection of OAD and a single intravenous injection of T cells via the tail vein (**Figures 6A**, **B** upper). On day 35 after tumor inoculation, 10^6^ PFU of OAD was administered intratumorally, followed by intravenous injection of 5 × 10^6^ T cells 5 days later. As expected, OAD alone and OAD combined with scrambled T cells showed moderate antitumor effects. By contrast, the combination of OAD and SpyCatcher CAR-T cells resulted in significant tumor suppression (**Figures 6C–E**). Flow cytometry was performed to analyze SpyTag-positive cells within the tumors (**Figure 6F**; **Supplementary Figure 8**). The combination treatment group showed a positivity rate of 3.95-12.2%, which was higher than that in the other treatment groups. Owing to extensive tumor necrosis, the tumor tissue contained abundant mucinous material resulting from necrosis, and cellularity was relatively sparse (**Figure 6G**). To allow for closer observation of OAD infection and T cell infiltration within the tumors, the treatment timeline was shortened (**Figure 6B** lower). CAR-T cells were administered two days after OAD injection, and tumors were harvested on day 38. Histological examination revealed patchy necrosis in the tumors, with areas positive for adenovirus and infiltrating T cells observed at the margins of these necrotic regions (**Figure 6H**; **Supplementary Figure 9**).

### Antitumor efficacy of combination therapy of OAD and SpyCatcher CAR-T cells in an immunocompetent murine syngeneic tumor model

To evaluate the therapeutic efficacy of the combination strategy in an immunocompetent setting, murine syngeneic tumor models were established. T cells were isolated from the spleens of C57BL/6 mice and transduced with a retroviral vector encoding the SpyCatcher CAR containing SpyCatcher, murine CD28 hinge, transmembrane domain and intracellular signaling domain, and a CD3 zeta intracellular signaling domain (**Supplementary Figures 10A, B**). The oncolytic virus expressing SpyTag-Ag85B retained replicative capacity and induced oncolysis in murine prostate cancer TRAMPC-1 cells (**Supplementary Figures 10C, D**).

**Figure 7:**
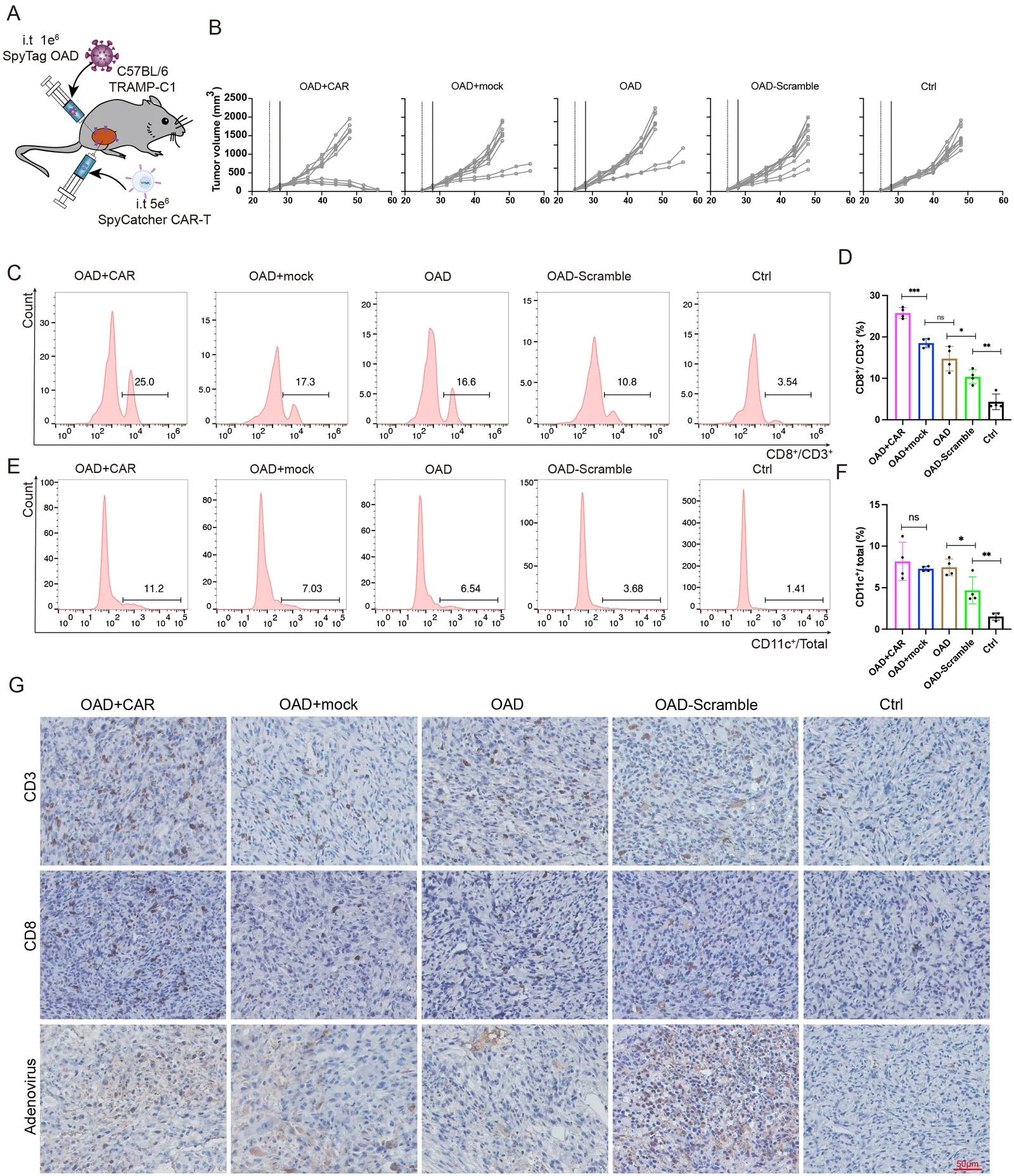
Antitumor efficacy of combination therapy of OAD and SpyCatcher CAR-T cells in an immunocompetent murine syngeneic tumor model. **(A)** Schematic diagram of the combination treatment with OAD-SpyTag oncolytic adenovirus and SpyCatcher CAR-T cells in an immunocompetent murine. Male C57BL/6 mice (n=10/group) were subcutaneously inoculated with 5 × 10^6^ TRAMP-C1 cells. Intratumoral injection of 1 × 10^6^ PFU OAD and 5 × 10^6^ SpyCatcher CAR-T cells or mock T cells were performed on days 25 and 28, respectively. **(B)** Tumor volume was measured every 3 days. On day 48, a subset of mice was sacrificed for tumor microenvironment analysis, while the remaining mice were monitored until day 56. **(C, D)** Quantification of percent cells positive for CD8, CD3 and CD11c **(E, F)** in TRAMP-C1 tumors from mice receiving the indicated treatments by flow cytometry. n=4 per group. **(G)** Immunohistochemical staining for CD3, CD8, and adenovirus (anti-Hexon) in tumor tissues.

Antitumor activity was assessed in a subcutaneous TRAMPC-1 tumor model in C57BL/6 mice. At day 25 post tumor inoculation, mice received an intratumoral injection of 1 × 10^6^ PFU OAD, followed by intratumoral administration of 5 × 10^6^ SpyCatcher CAR-T cells three days later (**Figure 7A**). The combination treatment induced complete tumor regression in 50% of mice, whereas tumor growth inhibition was observed in approximately 20% of mice treated with oncolytic adenovirus alone or in combination with control T cells (**Figure 7B**). To assess the contribution of Ag85B, an oncolytic adenovirus lacking Ag85B expression was included as a control. This group exhibited reduced antitumor activity compared to Ad-SpyTag-Ag85B (**Figure 7B**).

At day 48, tumors were harvested for analysis of the tumor microenvironment. Flow cytometric analysis showed increased frequencies of CD8^+^/CD3^+^ (**Figures 7C, D**; **Supplementary Figure 11**) and CD11c^+^ cells (**Figures 7E, F**; **Supplementary Figure 12**) in the combination treatment group. At the end of the experiment, the tumor-infiltrating CD3^+^ and CD8^+^ cells were endogenous murine T cells rather than the intratumorally injected SpyCatcher CAR-T cells (**Supplementary Figure 13**). Consistently, immunohistochemical staining demonstrated increased infiltration of CD3^+^ and CD8^+^ cells in tumors from mice receiving combination therapy (**Figure 7G**; **Supplementary Figure 14**). In contrast, the OAD-Scramble group exhibited lower proportions of CD3^+^, CD8^+^, and CD11c^+^ cells compared with the OAD group. Adenoviral staining in this group was predominantly localized to both the nucleus and cytoplasm. Through histological analysis (**Supplementary Figure 15**) and immunohistochemical staining for adenovirus (**Supplementary Figure 16**) of adjacent muscle and various organs, along with immunohistochemical staining for adenovirus, we found that the injected adenovirus did not accumulate in non-tumor tissues, and no adverse events were observed.

### Patient-derived prostate cancer organoids validate the translational potential of the spytag oad/spycatcher CAR-T platform

**Figure 8:**
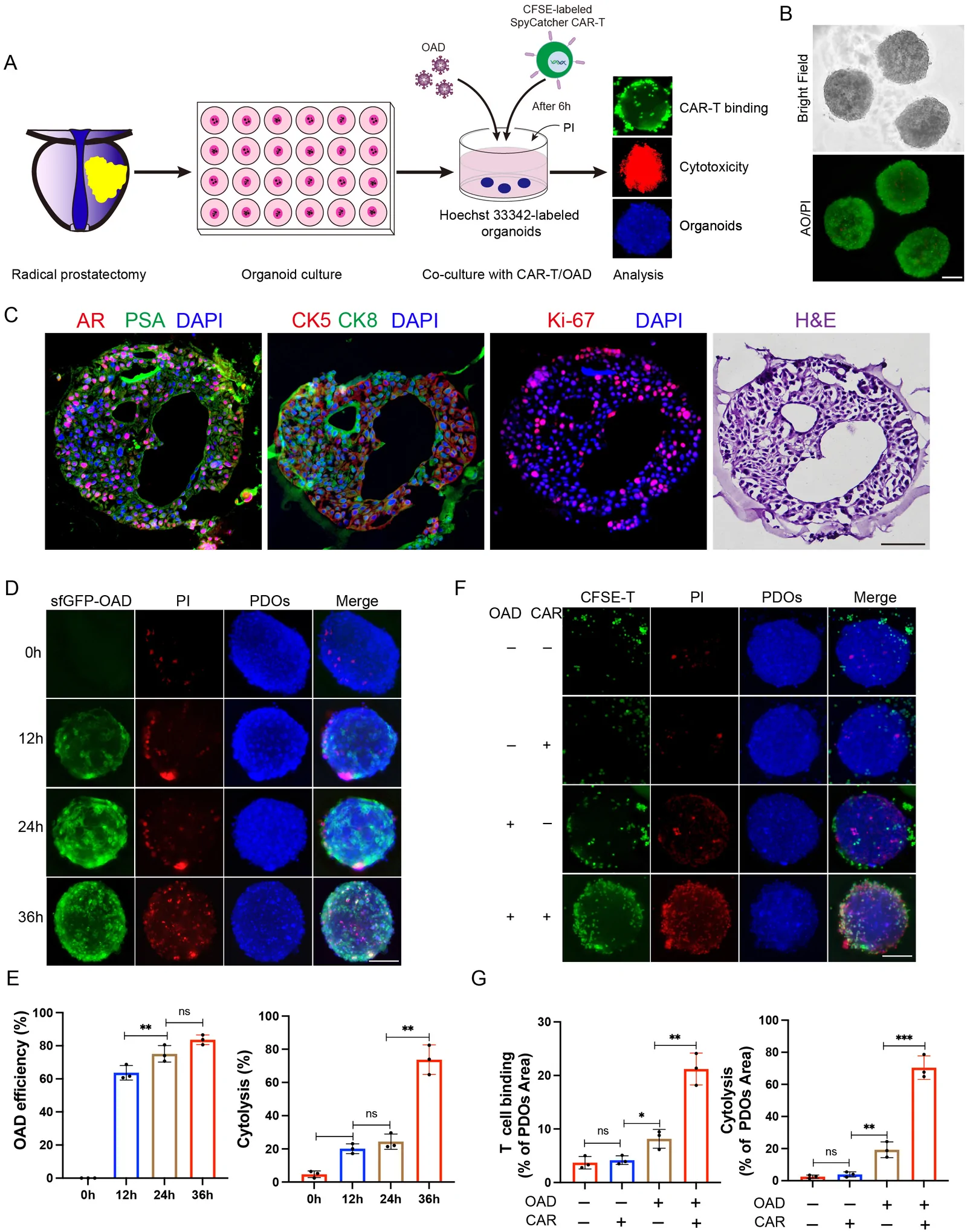
Patient-derived prostate cancer organoids validate the translational potential of the spytag oad/spycatcher CAR-T platform. **(A)** Schematic illustration of PDOs establishment, OAD infection, and SpyCatcher CAR-T-cell coculture assays. **(B)** Characterization of PDO viability by acridine orange/propidium iodide (AO/PI) staining. **(C)** Histological and phenotypic characterization of PDOs. Organoids were embedded in OCT, cryosectioned, and subjected to hematoxylin and eosin (H&E) staining and multiplex immunofluorescence staining. Representative images show expression of androgen receptor (AR), prostate-specific antigen (PSA), cytokeratin 5 (CK5), cytokeratin 8 (CK8), and Ki67. **(D)** Representative fluorescence images of PDOs infected with sfGFP-expressing oncolytic adenovirus (OAD-sfGFP) for 12, 24 and 36h. **(E)** Quantification of OAD infection efficiency and virus-mediated cytotoxicity in PDOs. **(F)** Representative fluorescence images of PDOs infected with Ag85B-SpyTag-expressing OAD and cocultured with SpyCatcher CAR-T cells. Organoids were labeled with Hoechst 33342 (blue), SpyCatcher CAR-T cells were labeled with CFSE (green), and dead cells were identified by PI staining (red). **(G)** Quantification of SpyCatcher CAR-T-cell binding and cytotoxicity toward PDOs following infection with Ag85B-SpyTag-expressing OAD. Scale bars, 100 μm.

To further evaluate the translational applicability of the SpyTag OAD/SpyCatcher CAR-T platform, we established patient-derived prostate cancer organoids (PDOs) from treatment-naïve radical prostatectomy specimens (**Figure 8A**). Acridine orange/propidium iodide (AO/PI) staining demonstrated that the organoids exhibited a typical spherical morphology with high viability, indicating successful establishment and maintenance of the cultures (**Figure 8B**). Histological and immunophenotypic characterization confirmed the prostate cancer origin of the organoids (**Figure 8C**).

To determine whether prostate cancer organoids were susceptible to OAD infection, PDOs were infected with an sfGFP-expressing OAD. sfGFP-positive cells were readily detectable as early as 12 h post-infection, accompanied by limited cell death (**Figure 8D**). By 36 h post-infection, sfGFP expression was observed throughout most organoids, with approximately 80% of the organoid area exhibiting sfGFP fluorescence, and a marked increase in dead cells was detected, indicating efficient viral propagation and oncolytic activity within the organoid model (**Figure 8E**).

Next, PDOs were infected with the Ag85B-SpyTag-expressing OAD and subsequently co-cultured with either SpyCatcher CAR-T cells or scrambled control T cells 6 h later (**Figure 8F**). After 24 h of co-culture, substantial accumulation of SpyCatcher CAR-T cells was observed around OAD-infected organoids. Importantly, extensive tumor cell death was detected within OAD-infected organoids in the SpyCatcher CAR-T-treated group, whereas scramble T cells showed minimal binding and cytotoxicity. These findings demonstrate that OAD-mediated SpyTag expression efficiently redirects SpyCatcher CAR-T cells to patient-derived prostate cancer organoids and enables potent antitumor activity in a clinically relevant PDOs model, supporting the translational potential of this strategy for prostate cancer treatment. (**Figure 8G**).

## Discussion

Our study addresses two major challenges in CAR-T cell therapy for solid tumors: tumor heterogeneity and the lack of tumor-specific antigens (40, 41). To overcome these limitations, we developed a universal therapeutic platform for solid tumors by combining an OAD with the SpyCatcher/SpyTag system. This approach enables tumor-selective labeling of tumor cells with Ag85B-SpyTag and subsequent covalent recognition by SpyCatcher CAR-T cells, thereby achieving efficient and specific tumor cell killing.

Oncolytic viruses provide a flexible platform for tumor-selective gene delivery and have been widely engineered to express immunomodulatory molecules or artificial antigens to enhance antitumor immunity and enable targeted therapies (42, 43). Adenoviruses are particularly attractive due to their safety profile, non-integrative nature, and large genetic capacity (44). In this study, we employed an E1B-55k-deleted adenovirus combined with an hTERT promoter-driven E1A system to achieve tumor-selective replication while minimizing off-target effects in normal tissues. To further potentiate antitumor efficacy, we engineered the virus to encode Ag85B, a major antigen derived from Mycobacterium tuberculosis (45, 46). Ag85B serves dual functions in our system. First, as a membrane-anchored scaffold, it facilitates the surface presentation of SpyTag and reduces steric hindrance for efficient recognition by SpyCatcher CAR-T cells. Second, as a highly immunogenic antigen, it enhances host immune activation against infected tumor cells (47, 48).

Consistent with this, we observed that Ag85B-expressing OAD induced more pronounced remodeling of the tumor microenvironment compared to the control virus in immunocompetent mice. Notably, immunohistochemical analysis revealed distinct viral distribution patterns. In tumors treated with Ag85B-negative OAD, viral signals were predominantly localized within the nucleus and cytoplasm of tumor cells, resembling the pattern observed in NSG mice. In contrast, Ag85B-expressing OAD resulted in viral signals primarily distributed within the tumor interstitium. We speculate that Ag85B expression promotes robust immune-mediated tumor cell killing, leading to the release of viral particles into the extracellular matrix and consequent interstitial localization. This interpretation is further supported by observations in NSG mice, where the lack of effective immune responses correlates with predominantly intracellular viral distribution.

These findings suggest that the spatial distribution of viral signals reflects both the host immune status and the immunostimulatory capacity of the OAD. In line with this concept, previous studies have shown that introducing non-self antigens, such as α1,3GT or sfGFP, can enhance immune recognition and enable redirected CAR-T cell cytotoxicity (49, 50). Together, our results support the concept of OAD-mediated “antigen delivery” as a generalizable strategy to overcome tumor heterogeneity and antigen loss by uniformly labeling tumor cells with targetable non-self markers.

The affinity between CAR-T cells and their target antigens is a critical determinant of therapeutic efficacy (51). High-avidity CAR-T cells can maintain function under low antigen density, thereby reducing tumor escape, but may also increase the risk of off-tumor toxicity due to recognition of low-level antigen expression in normal tissues (52, 53). Rather than further tuning affinity, our approach introduces a distinct mode of antigen recognition. By leveraging the SpyCatcher/SpyTag system, we establish covalent antigen engagement in CAR-T cells, enabling highly specific and irreversible binding to OAD-labeled tumor cells. This design represents a conceptual shift from conventional scFv-based recognition and provides a new framework for improving both specificity and functional stability of CAR-T cells.

Our strategy also differs from previously reported universal or adaptor CAR-T systems, including biotin-, leucine zipper-, SUPRA-, and convertible CAR-based approaches, which rely on reversible adaptor molecules to bridge CAR-T cells and target antigens (54, 55). These systems offer modularity and dose-controlled activity but generally require repeated adaptor administration and are limited by transient interactions. In contrast, the SpyCatcher/SpyTag system establishes irreversible covalent binding following OAD-mediated antigen delivery, thereby eliminating the need for continuous adaptor dosing while maintaining tumor-selective targeting.

However, the covalent nature of this interaction may also impose functional constraints. Limited dissociation after target engagement could impair serial killing capacity and lead to sustained signaling, potentially accelerating T cell exhaustion. This may partly explain the limited persistence of SpyCatcher CAR-T cells observed at later stages following intratumoral administration. Long-term persistence of SpyCatcher CAR-T cells should also be systematically evaluated in future studies, particularly under repeated antigen exposure. Future optimization of this platform will be required to improve CAR-T cell expansion and persistence. For example, strategies that promote T cell proliferation and maturation in lymphoid organs prior to tumor engagement may enhance antitumor efficacy while reducing premature exhaustion. Optimization of CAR design, cytokine support, or memory T-cell enrichment strategies may improve persistence while preserving the advantages of covalent antigen recognition.

Several limitations of this study should be considered. First, our findings are primarily based on mouse models, which may not fully recapitulate the complexity of human immune responses to OAD and CAR-T cell therapy. Second, our study mainly employed intratumoral administration of OAD, and the feasibility and efficacy of systemic delivery remain to be determined. Although intratumoral injection is clinically feasible for accessible lesions, including melanoma, head and neck cancer, and liver metastases, it is less suitable for disseminated or deeply located tumors. Systemic administration of OAD faces additional challenges, including rapid neutralization by pre-existing anti-adenovirus antibodies, hepatic sequestration, limited tumor extravasation, and inefficient intratumoral spread. Future studies may improve systemic delivery through capsid engineering, polymer shielding, cell carrier-based delivery, or regional vascular administration, thereby extending the applicability of this platform to metastatic disease. In addition, the uniformity and stability of OAD-mediated SpyTag expression within heterogeneous tumors require further investigation, as incomplete labeling may contribute to residual disease or tumor escape. Although OAD-mediated antigen delivery is expected to spatially restrict SpyTag expression to tumor cells, inadvertent viral infection of normal tissues or leakage of transgene expression could theoretically result in off-tumor recognition by SpyCatcher CAR-T cells. Potential immune-related toxicities, viral clearance, and long-term CAR-T cell persistence also warrant further evaluation. Notably, in immunocompetent mouse models, complete tumor regression was observed only in a subset of treated animals, while the majority exhibited limited therapeutic responses despite clear improvement in the tumor microenvironment. The mechanisms underlying this incomplete response remain unclear, but may reflect residual immunosuppressive pathways within the tumor. These findings suggest that combination strategies, particularly with immune checkpoint blockade, may be required to achieve more consistent and durable antitumor efficacy (56). Another potential limitation is the immunogenicity of the non-human components used in this platform. SpyCatcher/SpyTag originates from Streptococcus pyogenes, whereas Ag85B is derived from Mycobacterium tuberculosis. Future studies should evaluate anti-SpyCatcher, and anti-SpyTag immune responses in clinically relevant models and explore de-immunization or humanization strategies if necessary.

From a translational perspective, OAD-mediated antigen delivery provides a flexible strategy to expand the applicability of CAR-T cell therapy to solid tumors lacking suitable target antigens (57, 58). Both adenoviral vectors and CAR-T cell products have already entered clinical practice or advanced clinical development, providing an established manufacturing framework for this combination strategy. The SpyCatcher CAR construct can be generated using conventional CAR-T manufacturing workflows, whereas the engineered OAD can be produced using existing adenoviral vector production platforms, supporting scalability and Good Manufacturing Practice (GMP)-compatible manufacturing. Clinically, initial evaluation of this platform may be most suitable for patients with accessible solid tumors amenable to intratumoral injection, followed by optimization toward regional or systemic administration for metastatic disease. Furthermore, rational combinations with immune checkpoint blockade or other immunomodulatory approaches may further enhance therapeutic efficacy. Future studies should evaluate optimal delivery routes, including regional and systemic administration, as well as the safety and clinical feasibility of this combination strategy.

In summary, we developed a universal therapeutic platform that integrates OAD-mediated antigen delivery with covalent SpyCatcher CAR-T cell targeting. This approach enables tumor-selective labeling and efficient elimination of tumor cells while minimizing off-target effects. More broadly, this work establishes a new paradigm for programmable antigen targeting in CAR-T therapy and provides a promising strategy to overcome antigen heterogeneity and scarcity in solid tumors.

## Data Availability

The original contributions presented in the study are included in the article/**Supplementary Material**. Further inquiries can be directed to the corresponding authors.

## Glossary

AO/PI: acridine orange/propidium iodide
APC: allophycocyanin
BSA: bovine serum albumin
CFSE: carboxyfluorescein succinimidyl ester
CAR-T: Chimeric antigen receptor T
DMEM: Dulbecco’s Modified Eagle Medium
ECL: enhanced chemiluminescence
FACS: fluorescent activated cell sorting
GFP: green fluorescent protein
H&E: hematoxylin and eosin
HEPES: 4-(2-hydroxyethyl)-1-piperazineethanesulfonic acid
hTERT: human telomerase reverse transcriptase
IHC: immunohistochemistry
IFN-γ: interferon gamma
IL-2: interleukin-2
NFAT: Nuclear Factor of Activated T-cells
NSG: NOD scid gamma
OAD: oncolytic adenovirus
OV: Oncolytic viruses
OCT: optimal cutting temperature compound
PDOs: patient-derived organoids
PBS: phosphate-buffered saline
PFU: plaque-forming units
PEI: polyethylenimine
scFv: single-chain variable fragment
SC-sfGFP: SpyCatcher-sfGFP
ST-mCherry: SpyTag-mCherry
sfGFP: superfolder green fluorescent protein
TfR: transferrin receptor
TSA: tumor-specific antigens
MOI: multiplicity of infection
PD-1: programmed death-1
TIM-3: T-cell immunoglobulin and mucin domain 3
LAG-3: lymphocyte-activation gene 3.
