# Supplementary material for: A generalizable covalent car-t platform for solid tumors enabled by oncolytic adenovirus-delivered artificial antigens
Source: Front Immunol. 2026 Aug 26;17:1919208. doi: 10.3389/fimmu.2026.1919208 (PMC13553359; doi:10.3389/fimmu.2026.1919208)
Supplement: Supplementary file 1 [file Table1.docx]

Supporting Information

Supplement figure 1 SDS-PAGE gel electrophoresis with Coomassie blue staining. Lanes from left to right: Marker; SpyCatcher-sfGFP (SC-sfGFP, 42.4 kDa); SpyTag-mCherry (ST-mCherry, 30.4 kDa); SpyCatcher-sfGFP and SpyTag-mCherry mixed at a 1:1 mass ratio and incubated at room temperature for 10 min; SpyCatcher-sfGFP and SpyTag-mCherry mixed at a 3:1 mass ratio and incubated at room temperature for 10 min; SpyCatcher-sfGFP and SpyTag-mCherry mixed at a 1:3 mass ratio and incubated at room temperature for 10 min; SpyCatcher-mcherry (SC-mcherry, 42.3 kDa); SpyTag-sfGFP (SC-sfGFP, 30.7 kDa); SpyCatcher- mcherry and SpyTag- sfGFP mixed at a 1:1 mass ratio and incubated at room temperature for 10 min; Marker. Four fusion proteins were constructed using pET-28b(+) and purified using nickel columns.

Supplement figure 2 CD69 expression in six CAR-T cells post-target/off-target engagement. CAR +Target cell^ST+^: Jurkar cells expressed SpyCatcher CAR and engagement with target cells expression SpyTag; CAR +Target cell^ST-^: Jurkar cells expressed SpyCatcher CAR and engagement with wild type cells that did not expression SpyTag; CAR: Jurkar cells expressed SpyCatcher CAR and not engagement with other cells.

Supplement figure 3 Cytotoxic activity against SpyTag^+^ targets. Target cells expressing SpyTag incubated with CAR-T labeled with a cell proliferation staining reagent (Ex/Em=628/643) for 24 hours with E:T =1:1. After 24 h of incubation, the number of unlabeled cells was quantified by flow cytometry.

Supplement figure 4 Exhaustion profiling: PD-L1, TIM-3, and LAG-3 expression in CAR-T cells post-target engagement. (A) CAR-T cells were co-cultured with target cells at a 1:1 ratio for 24 hours, followed by re-stimulation with fresh target cells for an additional 24 hours. The expression of LAG-3, TIM-3, and PD-1 on T cells was then analyzed by flow cytometry (A). The mean fluorescence intensity (MFI) of LAG-3, TIM-3, and PD-1 was quantified (B). n = 3 per group.

Supplement figure 5 Dose-dependent transduction efficiency in PC3 cells 24 h post-infection, assessed by GFP expression (green) and adenoviral hexon protein detection.

Supplement figure 6 Time-course analysis of SpyTag delivery and adenoviral replication. Adenovirus was added to PC3 cells at different MOIs, and the expression of the target protein and the adenoviral protein, hexon, was detected 24, 48, and 72 hours.

Supplement figure 7 Flow cytometric analysis of CD8⁺ T cells expressing IFN-γ (upper) and Granzyme B (lower) following co-culture with PC3 cells infected with Ad-SpyTag-Ag85B at different multiplicities of infection (MOI).

Supplement figure 8 Single-cell suspensions were prepared from tumor tissues. SpyTag-positive tumor cells were labeled with SpyCatcher-sfGFP. Flow cytometry detected the proportion of positive cells.

Supplement figure 9 IHC staining for adenovirus and CD3 were performed on samples from the short-duration experiment.

Supplement figure 10 Synergistic cytotoxic effects of Ad-SpyTag-Ag85B and murine SpyCatcher CAR-T in vitro.

(A) Schematic representation of the murine SpyCatcher CAR construct, consisting of SpyCatcher, the murine CD28 hinge domain, murine CD28 transmembrane domain, the intracellular domain of murine CD28 as a costimulatory module, and the intracellular domain of murine CD3ζ. (B) Splenic T cells isolated from mice were transduced with retrovirus, and transduction efficiency was assessed by flow cytometry. (C) Dose-dependent transduction efficiency in TRAMP-C1 cells 24 h post-infection, assessed by Hexon expression. (D) Luciferase assay measuring MOI-dependent cytotoxicity mediated by murine SpyCatcher CAR-T cells (E:T = 1:1). *p < 0.05, **p < 0.01, ***p < 0.001, ****p < 0.0001.

Supplement figure 11 Proportions of CD3⁺ and CD8⁺ T cells in an immunocompetent murine syngeneic tumor model. Single-cell suspensions were prepared from TRAMP-C1 subcutaneous tumors following different treatments after mice were sacrificed, and CD3⁺ and CD8⁺ cells were analyzed by flow cytometry.

Supplement figure 12 Proportions of CD11c+ cells in an immunocompetent murine syngeneic tumor model. Single-cell suspensions were prepared from TRAMP-C1 subcutaneous tumors following different treatments after mice were sacrificed, and CD11c+ cells were analyzed by flow cytometry.

Supplement figure 13 IHC of SpyCatcher CAR-T cells in an immunocompetent murine syngeneic tumor model. SpyCatcher CAR-T cells were labeled using an anti-GFP tag antibody.

Supplement figure 14 IHC of CD3, CD8 and adenovirus positive cells in an immunocompetent murine syngeneic tumor model. adenovirus positive cells were labeled using an anti-hexon antibody.

Supplement figure 15 H&E staining of major organs and tumor-adjacent muscle tissues.

Supplement figure 16 Immunohistochemical staining for hexon in major organs and tumor-adjacent muscle tissues.

Data file S1 DNA sequence.

**TfR-Transferin receptor TM domain-Ag85B-MYC-SpyCatcher:**

atgatggatcaagctagatcagcattctctaacttgtttggtggagaaccattgtcatgtacccgggccagcctggctcggcaagtagatggcgataacagtcatgtggagatgaaacttgctgtagatgaagaagaaaatgctgacaataacacaaaggccaatgtcacaaaaccaaaaaggtgtagtggaagtatctgctatgggactattgctgtgatcgtctttttcttgattggatttatgattggctacttgggctatggaagcggaggatccgggggggcattcagtagaccaggcctgcccgtggagtatcttcaggtcccctctccgagcatgggccgcgatatcaaagtgcaattccagtctgggggcaataactcaccagctgtgtatttgctcgacggccttcgcgctcaggatgattataacggctgggatatcaatacacccgccttcgagtggtactaccaaagcggtctgtctatagtgatgcccgtgggtggccagagctccttttattctgattggtattcaccagcgtgtggtaaagctggctgccagacttacaaatgggagaccttcctcacaagcgaactgccacagtggttgtctgccaatagagccgtgaagcccacaggcagcgccgctatcggcctcagcatggccggcagctcagcaatgattctggctgcctaccatcctcagcaattcatttatgcaggcagtctgtcagccctcctggatccctcccaggggatgggcccatcactgattggattggcgatgggggacgctgggggctataaggctgccgatatgtggggaccctcttccgaccccgcttgggagcggaacgatccgacacagcagatccccaagctggtggccaacaatacacgcctgtgggtttattgcggcaacggaaccccgaatgagctggggggagcaaacatacctgccgaattccttgaaaatttcgtgcggagcagcaacctcaagttccaggacgcatacaacgcggctgggggacacaacgcagtgtttaatttccccccaaacgggacacacagctgggaatattggggcgcccaactcaacgcaatgaaaggagacctgcagagttctctcggggctggggaacaaaaactcatctcagaagaggatctgggttccggtggctcgggtggatccatggtgaccacactgtccggactgtctggagagcagggaccatccggcgacatgaccacagaggaggattctgccacacacatcaagttcagcaagagggacgaggacggaagagagctggcaggagcaaccatggagctgagggatagctccggcaagaccatcagcacatggatctccgacggccacgtgaaggatttctacctgtatcccggcaagtacacctttgtggagacagcagcaccagacggatatgaggtggcaacccctatcgagtttacagtgaacgaggacggacaggtgaccgtggatggagaggcaacagagggcgatgcacacaccggatctagcggatcttaa

**TfR-Transferin receptor TM domain-RFP -MYC-SpyTag:**

atgatggatcaagctagatcagcattctctaacttgtttggtggagaaccattgtcatgtacccgggccagcctggctcggcaagtagatggcgataacagtcatgtggagatgaaacttgctgtagatgaagaagaaaatgctgacaataacacaaaggccaatgtcacaaaaccaaaaaggtgtagtggaagtatctgctatgggactattgctgtgatcgtctttttcttgattggatttatgattggctacttgggctatggaagcggaggatccggggccagctccgaggatgtcatcaaagagtttatgagatttaaggtcaagatggagggaagcgtcaacggacacgagttcgagattgagggagaaggagaaggccggccttacgagggcacacaaaccgctaagctcaaggtcacaaaaggaggacccctccccttctcctgggatattctgagccctcagttccagtacggaagcaaagcctatgttaaacaccctgccgacatccctgactatctgaagctctccttccctgaaggcttcaagtgggagagattcatgaacttcgaggacggaggcgtggtgacagtcacacaagatagcaccctccaggacggagagtttatttataaggtgaaactcagaggaaccaacttcccctccgatggccctgtcatgcaaaaaaaaacaatgggatgggaagcctccaccgagagaatgtatcctgaggatggcgctctgaaaggcgaaattaaaatgagactgaaactcaaagacggaggacactacgatgccgaggtcaaaacaacctacaaggccaagaaacaagtgcagctgcctggcgcctacatgactgatattaaactcgacattatcagccataatggggactacaccatcgtggaacaatatgagagagctgagggcagacatagcacaggcgctggagaacaaaaactcatctcagaagaggatctgggttccggtggctcgggtggatcccgtggcgttcctcatattgttatggtggacgcctacaaacgctataaatcgggaggtggttcaggctaa

**pET28-sfGFP-SpyCatcher-His:**

atgggcagcagcatgcgtaaaggcgaagagctgttcactggtgtcgtccctattctggtggaactggatggtgatgtcaacggtcataagttttccgtgcgtggcgagggtgaaggtgacgcaactaatggtaaactgacgctgaagttcatctgtactactggtaaactgccggtaccttggccgactctggtaacgacgctgacttatggtgttcagtgctttgctcgttatccggaccatatgaagcagcatgacttcttcaagtccgccatgccggaaggctatgtgcaggaacgcacgatttcctttaaggatgacggcacgtacaaaacgcgtgcggaagtgaaatttgaaggcgataccctggtaaaccgcattgagctgaaaggcattgactttaaagaagacggcaatatcctgggccataagctggaatacaattttaacagccacaatgtttacatcaccgccgataaacaaaaaaatggcattaaagcgaattttaaaattcgccacaacgtggaggatggcagcgtgcagctggctgatcactaccagcaaaacactccaatcggtgatggtcctgttctgctgccagacaatcactatctgagcacgcaaagcgttctgtctaaagatccgaacgagaaacgcgatcatatggttctgctggagttcgtaaccgcagcgggcatcacgcatggtatggatgaactatacaaaggtggtggtggttctggtggtggtggttctgttactaccctgtctggtctgagcggcgaacaaggtccttccggtgatatgactaccgaagaggactctgccacccacatcaagttcagcaaacgtgatgaagacggtcgcgaactggctggtgcaaccatggaactgcgtgattctagcggtaagaccatctccacgtggattagcgacggccatgtgaaagatttctacctgtacccgggcaaatacaccttcgttgaaaccgctgccccggatggttacgaagtagcgacgccgatcgagttcaccgttaacgaggacggtcaggtaaccgtcgacggtgaagcaaccgaaggtgatgcacacaccggtggtggtggttctcatcatcaccaccatcatcaccaccatcattga

**pET28-sfGFP-SpyTag-His:**

atgggcagcagcatgcgtaaaggcgaagagctgttcactggtgtcgtccctattctggtggaactggatggtgatgtcaacggtcataagttttccgtgcgtggcgagggtgaaggtgacgcaactaatggtaaactgacgctgaagttcatctgtactactggtaaactgccggtaccttggccgactctggtaacgacgctgacttatggtgttcagtgctttgctcgttatccggaccatatgaagcagcatgacttcttcaagtccgccatgccggaaggctatgtgcaggaacgcacgatttcctttaaggatgacggcacgtacaaaacgcgtgcggaagtgaaatttgaaggcgataccctggtaaaccgcattgagctgaaaggcattgactttaaagaagacggcaatatcctgggccataagctggaatacaattttaacagccacaatgtttacatcaccgccgataaacaaaaaaatggcattaaagcgaattttaaaattcgccacaacgtggaggatggcagcgtgcagctggctgatcactaccagcaaaacactccaatcggtgatggtcctgttctgctgccagacaatcactatctgagcacgcaaagcgttctgtctaaagatccgaacgagaaacgcgatcatatggttctgctggagttcgtaaccgcagcgggcatcacgcatggtatggatgaactatacaaaggtggtggtggttctggtggtggtggttctggtggtggtggttctcgtggcgttcctcatattgttatggtggacgcctacaaacgctataaaggtggtggtggttctcatcatcaccaccatcatcaccaccatcattga

**8/28/28 CAR**

atgcttctcctggtgacaagccttctgctctgtgagttaccacacccagcattcctcctgatcccacatatggtgaccacactgtccggactgtctggagagcagggaccatccggcgacatgaccacagaggaggattctgccacacacatcaagttcagcaagagggacgaggacggaagagagctggcaggagcaaccatggagctgagggatagctccggcaagaccatcagcacatggatctccgacggccacgtgaaggatttctacctgtatcccggcaagtacacctttgtggagacagcagcaccagacggatatgaggtggcaacccctatcgagtttacagtgaacgaggacggacaggtgaccgtggatggagaggcaacagagggcgatgcacacaccgtcgacttcgtaccagtttttctccctgccaaaccgaccaccacccccgccccacgcccaccaacaccagcccccactatcgcatcacagccgcttagtctcaggcccgaggcttgtagaccagctgccggtggggctgttcacacccggggtcttgacttcgcttgtgacttctgggttcttgtagtcgtcggaggcgtgttggcatgttactccctgctggtcactgtggccttcataatcttctgggtccggtctaagcggtccagacttctccactctgactacatgaacatgacccccaggagaccagggcccacgagaaagcattatcagccatatgcgccacctcgcgatttcgccgcataccgctccagagtgaagttcagcaggagcgcagacgcccccgcgtaccagcagggccagaaccagctctataacgagctcaatctaggacgaagagaggagtacgatgttttggacaagagacgtggccgggaccctgagatggggggaaagccgcagagaaggaagaaccctcaggaaggcctgtacaatgaactgcagaaagataagatggcggaggcctacagtgagattgggatgaaaggcgagcgccggaggggcaaggggcacgatggcctttaccagggtctcagtacagccaccaaggacacctacgacgcccttcacatgcaggccctgccccctcgctaa

**28/28/28 CAR**

atgcttctcctggtgacaagccttctgctctgtgagttaccacacccagcattcctcctgatcccacatatggtgaccacactgtccggactgtctggagagcagggaccatccggcgacatgaccacagaggaggattctgccacacacatcaagttcagcaagagggacgaggacggaagagagctggcaggagcaaccatggagctgagggatagctccggcaagaccatcagcacatggatctccgacggccacgtgaaggatttctacctgtatcccggcaagtacacctttgtggagacagcagcaccagacggatatgaggtggcaacccctatcgagtttacagtgaacgaggacggacaggtgaccgtggatggagaggcaacagagggcgatgcacacaccgtcgacattgaagttatgtatcctccttacctagacaatgagaagagcaatggaaccattatccatgtgaaagggaaacacctttgtccaagtcccctatttcccggaccttctaagcccttttgggtgctggtggtggttggtggagtcctggcttgctatagcttgctagtaacagtggcctttattattttctgggtgaggagtaagaggagcaggctcctgcacagtgactacatgaacatgactccccgccgccccgggcccacccgcaagcattaccagccctatgccccaccacgcgacttcgcagcctatcgctccagagtgaagttcagcaggagcgcagacgcccccgcgtaccagcagggccagaaccagctctataacgagctcaatctaggacgaagagaggagtacgatgttttggacaagagacgtggccgggaccctgagatggggggaaagccgcagagaaggaagaaccctcaggaaggcctgtacaatgaactgcagaaagataagatggcggaggcctacagtgagattgggatgaaaggcgagcgccggaggggcaaggggcacgatggcctttaccagggtctcagtacagccaccaaggacacctacgacgcccttcacatgcaggccctgccccctcgctaa

**8/8/28 CAR**

atgcttctcctggtgacaagccttctgctctgtgagttaccacacccagcattcctcctgatcccacatatggtgaccacactgtccggactgtctggagagcagggaccatccggcgacatgaccacagaggaggattctgccacacacatcaagttcagcaagagggacgaggacggaagagagctggcaggagcaaccatggagctgagggatagctccggcaagaccatcagcacatggatctccgacggccacgtgaaggatttctacctgtatcccggcaagtacacctttgtggagacagcagcaccagacggatatgaggtggcaacccctatcgagtttacagtgaacgaggacggacaggtgaccgtggatggagaggcaacagagggcgatgcacacaccgtcgacttcgtaccagtttttctccctgccaaaccgaccaccacccccgccccacgcccaccaacaccagcccccactatcgcatcacagccgcttagtctcaggcccgaggcttgtagaccagctgccggtggggctgttcacacccggggtcttgacttcgcttgtgacatatacatctgggctccacttgctggaacatgcggtgttctgcttctgagcctcgttatcacattctgggtccggtctaagcggtccagacttctccactctgactacatgaacatgacccccaggagaccagggcccacgagaaagcattatcagccatatgcgccacctcgcgatttcgccgcataccgctccagagtgaagttcagcaggagcgcagacgcccccgcgtaccagcagggccagaaccagctctataacgagctcaatctaggacgaagagaggagtacgatgttttggacaagagacgtggccgggaccctgagatggggggaaagccgcagagaaggaagaaccctcaggaaggcctgtacaatgaactgcagaaagataagatggcggaggcctacagtgagattgggatgaaaggcgagcgccggaggggcaaggggcacgatggcctttaccagggtctcagtacagccaccaaggacacctacgacgcccttcacatgcaggccctgccccctcgctaa

**8/8/BB CAR**

atgcttctcctggtgacaagccttctgctctgtgagttaccacacccagcattcctcctgatcccacatatggtgaccacactgtccggactgtctggagagcagggaccatccggcgacatgaccacagaggaggattctgccacacacatcaagttcagcaagagggacgaggacggaagagagctggcaggagcaaccatggagctgagggatagctccggcaagaccatcagcacatggatctccgacggccacgtgaaggatttctacctgtatcccggcaagtacacctttgtggagacagcagcaccagacggatatgaggtggcaacccctatcgagtttacagtgaacgaggacggacaggtgaccgtggatggagaggcaacagagggcgatgcacacaccgtcgacttcgtaccagtttttctccctgccaaaccgaccaccacccccgccccacgcccaccaacaccagcccccactatcgcatcacagccgcttagtctcaggcccgaggcttgtagaccagctgccggtggggctgttcacacccggggtcttgacttcgcttgtgacatatacatctgggctccacttgctggaacatgcggtgttctgcttctgagcctcgttatcacaaaacgcggaaggaagaagctcctctacatatttaagcaacccttcatgcgccctgtgcagacaacgcaggaagaagatggatgctcctgtcgcttccctgaagaagaggaaggaggttgtgagcttagagtgaagttcagcaggagcgcagacgcccccgcgtaccagcagggccagaaccagctctataacgagctcaatctaggacgaagagaggagtacgatgttttggacaagagacgtggccgggaccctgagatggggggaaagccgcagagaaggaagaaccctcaggaaggcctgtacaatgaactgcagaaagataagatggcggaggcctacagtgagattgggatgaaaggcgagcgccggaggggcaaggggcacgatggcctttaccagggtctcagtacagccaccaaggacacctacgacgcccttcacatgcaggccctgccccctcgctaa

**8/8/28BB CAR**

atgcttctcctggtgacaagccttctgctctgtgagttaccacacccagcattcctcctgatcccacatatggtgaccacactgtccggactgtctggagagcagggaccatccggcgacatgaccacagaggaggattctgccacacacatcaagttcagcaagagggacgaggacggaagagagctggcaggagcaaccatggagctgagggatagctccggcaagaccatcagcacatggatctccgacggccacgtgaaggatttctacctgtatcccggcaagtacacctttgtggagacagcagcaccagacggatatgaggtggcaacccctatcgagtttacagtgaacgaggacggacaggtgaccgtggatggagaggcaacagagggcgatgcacacaccgtcgacttcgtaccagtttttctccctgccaaaccgaccaccacccccgccccacgcccaccaacaccagcccccactatcgcatcacagccgcttagtctcaggcccgaggcttgtagaccagctgccggtggggctgttcacacccggggtcttgacttcgcttgtgacatatacatctgggctccacttgctggaacatgcggtgttctgcttctgagcctcgttatcacattctgggtccggtctaagcggtccagacttctccactctgactacatgaacatgacccccaggagaccagggcccacgagaaagcattatcagccatatgcgccacctcgcgatttcgccgcataccgctccaaacgcggaaggaagaagctcctctacatatttaagcaacccttcatgcgccctgtgcagacaacgcaggaagaagatggatgctcctgtcgcttccctgaagaagaggaaggaggttgtgagcttagagtgaagttcagcaggagcgcagacgcccccgcgtaccagcagggccagaaccagctctataacgagctcaatctaggacgaagagaggagtacgatgttttggacaagagacgtggccgggaccctgagatggggggaaagccgcagagaaggaagaaccctcaggaaggcctgtacaatgaactgcagaaagataagatggcggaggcctacagtgagattgggatgaaaggcgagcgccggaggggcaaggggcacgatggcctttaccagggtctcagtacagccaccaaggacacctacgacgcccttcacatgcaggccctgccccctcgctaa
